# ARG2 drives osteoblast senescence by repressing mitophagy via the IκB–NF-κB–TXN2 signaling axis: implications for age-associated osteoporosis

**DOI:** 10.7150/thno.129818

**Published:** 2026-09-18

**Authors:** Jinli Li, Liyao Zhang, Yuanyuan Ren, Junxuan Wang, Pan Zhang, Qianran Shen, Yaqi Wang, Yi Yu, Zi Cheng, Qiang Wang, Haiying Wang, Yuyan Xiong

**Affiliations:** 1 Key Laboratory of Resource Biology and Biotechnology in Western China, Ministry of Education, Faculty of Life Sciences and Medicine, College of Life Sciences, Northwest University, Xi'an, Shaanxi, China.; 2 School of Medicine, Northwest University, Xi’an, Shaanxi 710069, China.; 3 Department of Orthopaedics, Shenmu Hospital, The Affiliated Shenmu Hospital of Northwest University, Guangming Road, Shenmu, China.; 4 Department of Science and Education, Shenmu Hospital, The Affiliated Shenmu Hospital of Northwest University, Shenmu, China.

**Keywords:** Arginase 2, senile osteoporosis, osteoblast senescence, mitophagy, mitochondrial oxidative stress

## Abstract

**Rationale:**

Age-associated osteoporosis (AAOP) is characterized by progressive bone loss that can be driven by impaired osteoblast function and increased cellular senescence. However, the molecular mechanisms driving osteoblast senescence during aging remain incompletely understood. In this study, we aim to investigate the role of arginase 2 (ARG2) and its underlying mechanisms in osteoblast senescence and AAOP.

**Methods:**

AAOP were assessed in D-galactose-induced aging mice, naturally aged mice, and osteoblast-specific ARG2 knockout mice. *In vitro*, osteoblast senescence was induced by hydrogen peroxide exposure. Bone phenotypes, cellular senescence, mitochondrial function, and mitophagy were examined using histological, molecular, and imaging techniques. Luciferase reporter assays, co-immunoprecipitation (Co-IP), and pharmacological or genetic approaches were employed for molecular mechanistic investigations.

**Results:**

We observed that ARG2 expression was markedly elevated in senescent osteoblasts and aged mice with osteoporosis. Osteoblast-specific deletion of ARG2 alleviated age-related bone loss, improved trabecular bone microarchitecture, and reduced osteoblast senescence *in vivo*. Mechanistically, ARG2 promoted mitochondrial oxidative stress and impaired mitophagy, as evidenced by inhibition of the PINK1/Parkin/LC3 pathway and accumulation of p62. Furthermore, we revealed that ARG2 activated IκBα/NF-κB signaling, leading to HDAC9-mediated transcriptional repression of thioredoxin 2 (TXN2), a key mitochondrial antioxidant. Restoration of TXN2 expression, antioxidant treatment, or blockade of NF-κB/HDAC9 signaling rescued mitophagy, attenuated osteoblast senescence, and restored osteogenic function. Moreover, pharmacological inhibition of ARG2 using S-(2-boronoethyl)-L-cysteine (BEC) significantly suppressed osteoblast senescence and protected against age-related osteoporosis.

**Conclusion:**

ARG2 plays a pivotal role in osteoblast senescence and the development of AAOP by inducing mitochondrial oxidative stress and mitophagy dysfunction through the IκBα/NF-κB/HDAC9/TXN2 signaling axis. Targeting ARG2 may offer a promising therapeutic approach for AAOP prevention and treatment.

## Introduction

Age-associated osteoporosis (AAOP), character-rized by progressive bone loss and increased fracture risk, is a major public health concern affecting millions worldwide, particularly the elderly population 1. AAOP arises from an imbalance in bone remodeling, whereby osteoclast-mediated bone resorption exceeds osteoblast (OB)-mediated bone formation, leading to reduced bone mass and impaired bone microarchitecture 2. Cellular senescence, a state of irreversible arrest accompanied by functional decline, has emerged as a critical contributor to age-related bone loss 3. In particular, OBs senescence impairs bone formation and disrupts the bone remodeling process, in turn exacerbating AAOP 4. Notably, genetic and pharmacological clearance of senescent cells has been demonstrated to enhance bone formation and improve skeletal health in aged mice 5, 6. With the continued expansion of the aging population, the incidence of AAOP is expected to rise substantially, imposing a growing socioeconomic burden worldwide 7. As such, therapeutic strategies targeting osteoblast senescence have emerged as a promising approach for the treatment of AAOP 8, 9. Understanding the molecular mechanisms that drive osteoblast senescence is therefore essential for the development of effective therapies against AAOP.

Arginase 2 (ARG2) is a mitochondrial enzyme that catalyzes the hydrolysis of arginine to urea and ornithine 10, 11. Recent studies have implicated its important roles in modulating cellular senescence, oxidative stress, and age-related diseases 12. In the cardiovascular system, ARG2 has been revealed to promote reactive oxygen species (ROS) production, thereby leading to mitochondrial dysfunction and accelerated cellular aging. In endothelial cells (ECs), ARG2 drives the senescence of ECs through promotion of oxidative stress 13, and suppression of endothelial autophagy 14. In human vascular smooth muscle cells (VSMCs), ARG2 has been demonstrated to trigger VSMCs senescence through mechanisms involving mitochondrial dysfunction 12. Given the established roles of oxidative stress and mitochondrial impairment in osteoblast dysfunction and defective bone formation 15, ARG2 may serve as an important regulator of AAOP. However, the role of ARG2 in osteoblast senescence and AAOP remains largely unexplored.

Mitophagy, a selective form of autophagy that facilitates the removal of damaged mitochondria, plays a pivotal role in maintaining mitochondrial quality and cellular homeostasis. Impairment of mitophagy has been implicated in advanced oxidation protein product (AOPP)-induced osteoblast apoptosis and has been involved in bone loss, microarchitectural deterioration, and reduced bone mineral density (BMD) 16. These findings prompted us to investigate whether ARG2 contributes to osteoblast senescence through disruption of mitochondrial homeostasis and mitophagy during aging. In this study, we identify ARG2 as an important modulator of osteoblast senescence and AAOP. Our findings uncover a previously unrecognized axis that drives osteoblast senescence and bone loss, providing novel insight into the pathogenesis of AAOP and highlighting ARG2 as a promising therapeutic target for the prevention and treatment of AAOP.

## Materials and Methods

### Animals

All animal experiments in this study were approved by the Committee on Animal Experimentation and the Laboratory Animal Care and Use Committee of Northwest University. To model age-related osteoporosis, D-galactose (D-gal) administration was widely used to induce accelerated aging in mice, mimicking physiological changes observed in natural aging, including oxidative stress and bone loss 17. All the mice were randomly divided into ten groups containing twelve mice per group and received the following treatments: group 1, daily subcutaneous injection of physiological saline solution; group 2, daily subcutaneous injection of D-galactose (1000 mg/kg/day, 12 weeks) (Macklin, shanghai, China); group 3, male C57BL/6 mice of 20 months were arranged as old mice; group 4, daily subcutaneous injection of BEC (2.3 mg/kg/day, 6 weeks) (Aladdin, shanghai, China) together with injection of D-galactose (1000 mg/kg/day, 12 weeks); group 5, ARG2^flox/flox^ male C57BL/6 mice (Cyagen, Suzhou, China) of 2 months; group 6, osteoblastic-ARG2-specific knockout male mice (Cyagen, Suzhou, China) of 2 months; group 7, ARG2^flox/flox^ male C57BL/6 mice of 2 months together with injection of D-galactose (1000 mg/kg/day, 12 weeks); group 8, osteoblastic-ARG2-specific knockout male mice of 2 months together with injection of D-galactose (1000 mg/kg/day, 12 weeks); group 9, daily subcutaneous injection of D-galactose (1000 mg/kg/day, 12 weeks) and additionally received weekly tail-vein injections of TXN2-overexpressing lentivirus (EF1α-TXN2; 200 μL per injection) beginning at week 8; and group 10, daily subcutaneous injection of BEC (2.3 mg/kg/day, 6 weeks) (Aladdin, shanghai, China) to male C57BL/6 mice of 18 months. Blood samples were collected from the eyeball after anesthesia and centrifuged for plasma. The femurs were collected and fixed in 4% paraformaldehyde or frozen in liquid nitrogen for subsequent analysis. ARG2 conditional knockout mice (ARG2^flox/flox^; *Oc-Cre*^+^ mice) on a C57BL/6 background were generated using the Cre–loxP system. The ARG2^flox/flox^ allele (Cyagen, Suzhou, China; S-CKO-17785) was generated using a previously described conditional targeting strategy in which the conditional ARG2 region was flanked by loxP sites 18. ARG2^flox/flox^ mice were crossed with Osteocalcin-*Cre* (Oc-*Cre*) mice, in which Cre recombinase is driven by the osteocalcin promoter to mediate recombination predominantly in mature osteoblast-lineage cells 19. Briefly, female ARG2^flox/flox^ mice were first crossed with male mice carrying the *Oc*-*Cre* allele to generate ARG2^flox/+^; *Oc-Cre* mice, which were then mated again with ARG2^flox/flox^ mice to obtain ARG2^flox/flox^; *Oc-Cre* mice. Genotyping was performed by PCR. Genomic DNA from mouse tails was extracted, and PCR was performed using the KAPA mouse genotyping kit (Kapa Biosystems, Wilmington, USA) according to the manufacturer's instructions. PCR primers used were: 5’-TATCTCTGGATTTCATGTCCAACG-3’ (forward) and 5’-TAATTACCTGGGACAAAGTGGTGA (reverse) for loxP sequence, 5’-CTGCATATAAATTCTGGCTGGC-3’ (forward) and 5’-GAACATCTTCAGGTTCTGCGGGA-3’ (reverse) for *Oc-Cre*. All animal experiments were conducted with the approval of the Animal Ethics Review Committee of Northwest University (NWU-AWC-20242609M) and carried out in accordance with the guidelines from EU Directive 2010/63/EU.

### Micro-CT analysis

All micro-computed tomography (micro-CT) procedures were performed in accordance with the guidelines of the American Society for Bone and Mineral Research (ASBMR) 20. Briefly, femurs were harvested from mice, fixed in 4% paraformaldehyde for 48 h, and stored until analysis. Bone microarchitecture was evaluated using a high-resolution *ex vivo* micro-CT scanner (SkyScan 1276, Bruker, Kontich, Belgium). Quantitative analyses were conducted in the distal femoral metaphysis and midshaft femoral diaphysis. Three-dimensional (3D) reconstructions and morphometric measurements were generated from two-dimensional (2D) scan data using CTAn software (Bruker, Kontich, Belgium). For trabecular bone, the following morphometric parameters were evaluated: volumetric bone mineral density (vBMD), bone volume fraction (BV/TV), trabecular number (Tb.N), trabecular separation (Tb.Sp), and structure model index (SMI). For cortical bone, volumetric bone mineral density (vBMD), bone volume fraction (BV/TV), and cortical thickness (Ct.Th) were assessed at the femoral midshaft. All image acquisition and analyses were performed in a blinded manner.

### Calcein double labeling

Calcein (#C7600) was purchased from Solarbio (Beijing, China). Mice received intraperitoneal injections of calcein (15 mg/kg body weight) at 10 and 3 days prior to euthanasia. Following sacrifice, femurs were harvested, fixed, and sectioned for histological analysis. Calcein-labeled surfaces were visualized using a fluorescence microscope, and the mineral apposition rate (MAR) was determined according to a previously established protocol 21.

### Measurement of serum biomarkers

Mice were fasted for at least 12 h prior to euthanasia. Blood samples were collected and allowed to clot at room temperature for 1 h, followed by centrifugation at 4,000 rpm for 15 min at 4 °C. The resulting serum was collected and stored at -80 °C until analysis. Serum levels of procollagen type I N-terminal propeptide (PINP) and C-terminal telopeptide of type I collagen (CTX-I) were quantified using commercially available enzyme-linked immunosorbent assay (ELISA) kits (YOBIBIO, Shanghai, China) according to the manufacturer’s instructions. All measurements were performed in a blinded manner.

### Hematoxylin and eosin (H&E) staining

Femurs were harvested, fixed, and decalcified in 10% ethylenediaminetetraacetic acid (EDTA) at 4 °C for 10-15 days before paraffin embedding. Paraffin-embedded bone sections (4 μm thick) were deparaffinized, rehydrated, and rinsed in distilled water. Sections were then stained with hematoxylin solution (Yuanye, Shanghai, China) for 10 min, differentiated in hydrochloric acid ethanol for 30 s, rinsed thoroughly with water, and counterstained with eosin solution. After dehydration and mounting, histological images were acquired and analyzed. All histological evaluations were conducted in a blinded manner.

### Cell culture, osteogenic induction, and treatment

Murine pre-osteoblastic MC3T3-E1 cells (RRID: CVCL_0409) were obtained from the Committee of Type Culture Collection, Chinese Academy of Sciences (Beijing, China). All *in vitro* experiments were conducted using cells between passages 5 and 10. Cells were cultured in α-Minimum Essential Medium (α-MEM; Pricella, Wuhan, China) supplemented with 10% fetal bovine serum (FBS; ExCell Bio, Suzhou, China) and 1% penicillin-streptomycin (Beyotime, Shanghai, China) and maintained in a humidified incubator at 37 °C with 5% CO₂. Cells were routinely subcultured upon reaching 70-80% confluence, and the culture medium was replaced every 2 days. Primary bone marrow mesenchymal stem cells (BMSCs) were isolated and cultured as previously described 22. Briefly, femurs and tibiae were aseptically harvested from 6-week-old C57BL/6 mice. Bone marrow was flushed from the long bones using phosphate-buffered saline (PBS), and the remaining bone fragments were digested with type II collagenase (Worthington Biochemical Corp, USA). The digested bone fragments were cultured in α-MEM supplemented with 10% FBS and 1% penicillin-streptomycin at 37 °C in a humidified atmosphere containing 5% CO₂. After 48 h, released cells were removed, and the remaining bone fragments were further cultured to allow migration and expansion of fibroblast-like BMSCs. Non-adherent cells were discarded, and adherent cells were expanded until approximately 80% confluence, which was designated as passage 1 (P1). Passage 3 (P3) BMSCs were used for all subsequent experiments. Primary bone marrow monocytes (BMMs) were isolated and cultured in complete α-MEM containing 10% FBS, 2 mM L-glutamine, 1% penicillin-streptomycin, and macrophage colony-stimulating factor (M-CSF; 25 ng/mL). All cultures were maintained at 37 °C in a humidified incubator with 5% CO₂. Osteoclast differentiation was induced by treatment with receptor activator of nuclear factor-κB ligand (RANKL; 10 ng/mL, R&D Systems) and M-CSF (25 ng/mL) as previously described 23.

For osteogenic induction of MC3T3-E1 cells, the growth medium was supplemented with 10 mM β-glycerophosphate disodium salt hydrate (Macklin, Shanghai, China) and 50 μM L-ascorbic acid (Aladdin, Shanghai, China). For osteogenic differentiation of BMSCs, cells were cultured in α-MEM containing 10% FBS, 10 nM dexamethasone (Sigma-Aldrich, D4902, USA), 10 mM β-glycerophosphate, and 50 μM L-ascorbic acid. To establish an oxidative stress-induced senescence model, MC3T3-E1 cells were exposed to increasing concentrations of H₂O₂ (0-600 μM) for 24 h. Based on the dose-response results, 200 μM H₂O₂ was selected for subsequent experiments. For pharmacological interventions, MC3T3-E1 cells were pretreated for 24 h with rapamycin (100 nM; MedChemExpress, Shanghai, China), N-acetyl-L-cysteine (NAC; 500 μM; Aladdin, Shanghai, China), Mito-TEMPO (50 μM; Aladdin, Shanghai, China), BAY11-7082 (10 nM; Aladdin, Shanghai, China), iohexol (0-75 mg/mL; Aladdin, Shanghai, China), or S-(2-boronoethyl)-L-cysteine (BEC; 200 μM; Aladdin, Shanghai, China), followed by co-treatment with H₂O₂ (200 μM) for an additional 24 h. For mitophagic flux analysis, cells were treated with bafilomycin A1 (Baf A1; 100 nM; Solarbio, Beijing, China) for 4 h before sample collection.

### Senescence-associated β-galactosidase (SA-β-gal) staining assay

Cellular senescence was evaluated using a Senescence β-Galactosidase Staining Kit (Beyotime, Shanghai, China) according to the manufacturer’s instructions. Briefly, cells subjected to different treatments were washed with phosphate-buffered saline (PBS), fixed, and incubated with β-galactosidase staining solution at 37 °C overnight. Senescent cells were identified by the presence of blue staining under an inverted microscope. The percentage of SA-β-gal-positive cells was quantified by counting positively stained cells in randomly selected fields. All experiments were performed independently in triplicate.

### Western blot

Cultured cells were washed three times with ice-cold PBS and lysed in RIPA buffer. The lysates were centrifuged at 13,800 × g for 30 min at 4 °C, and the supernatants were collected for protein analysis. Protein concentrations were determined using a BCA Protein Assay Kit (Seven, Beijing, China). Equal amounts of protein were separated by 8-15% SDS-polyacrylamide gel electrophoresis (SDS-PAGE) and transferred onto polyvinylidene fluoride (PVDF) membranes. After blocking, the membranes were incubated sequentially with primary and appropriate horseradish peroxidase-conjugated secondary antibodies. Protein bands were detected using enhanced chemiluminescence reagents (ABclonal, Wuhan, China). The following primary antibodies were used: anti-p62/SQSTM1 (18420-1-AP), anti-LC3 (14600-1-AP), anti-p21 (10355-1-AP), anti-TXN2 (13089-1-AP), anti-CD44 (60224-1-Ig), and anti-RUNX2 (20700-1-AP) (all from Proteintech, Wuhan, China); anti-IκBα (#9242) and anti-phospho-IκBα (Ser32) (#2859) (Cell Signaling Technology, Danvers, MA, USA); anti-p53 (A21630), anti-ARG2 (A6355), anti-HDAC9 (A1516), anti-NF-κB p65/RelA (A19653), anti-CD34 (A13929), anti-OCN (A6205), and anti-β-actin (AC026) (ABclonal, Wuhan, China). Band intensities were quantified using Image J software and normalized to β-actin as an internal loading control.

### Immunofluorescence staining

MC3T3-E1 cells were cultured onto coverslips for 24 h. The cells were then washed with ice-cold PBS and fixed with 4% paraformaldehyde for 30 min at 4 °C. After three washes with PBS, the cells were blocked with 5% bovine serum albumin (BSA) in TBST for 30 min at room temperature to reduce nonspecific binding. The cells were then incubated with the indicated primary antibodies overnight at 4 °C. Following PBS washes, the cells were incubated with donkey anti-rabbit or anti-mouse IgG secondary antibodies (1:100) for 30 min at room temperature. Nuclei were counterstained with DAPI for 5 min. After washing with PBS, fluorescence images were acquired using a confocal laser scanning microscope (Leica, Mannheim, Germany).

### Total RNA extraction and qPCR analysis

Total RNA was extracted from cells or tissues using the RNA isolator Total RNA Extraction Reagent (Solarbio, Beijing, China). RNA concentration and purity (A260/280 ratio) were measured using a NanoDrop ND2000 spectrophotometer (KAIAO, Shenzhen, China). cDNA synthesis was performed using PrimeScript™ Reverse Transcriptase MasterMix (ABclonal, Wuhan, China). Quantitative PCR (qPCR) was carried out using a QuantStudio™ Dx Real-Time PCR System (ABclonal, Wuhan, China). Relative mRNA expression levels were calculated using the 2^⁻ΔΔCT^ method and normalized to GAPDH. All experiments were performed in triplicate.

### ALP activity assay and Alizarin red staining

For the ALP activity assay, cells were lysed using 1% Triton X-100 (Solarbio, Beijing, China) at 37 °C for 2 h after 3, 7, and 14 days of culture. ALP activity was measured using an ALP assay kit (Solarbio, Beijing, China). Total protein content was determined using a BCA protein assay kit, and ALP activity was normalized to protein concentration. For Alizarin Red S staining, MC3T3-E1 cells and primary osteoblasts were seeded in 3.5-cm culture dishes and maintained under osteogenic conditions for 21 days. Cells were washed twice with PBS and fixed with 4% paraformaldehyde at room temperature for 30 min. After fixation, cells were washed three times with PBS and stained with 0.2% Alizarin Red S (pH 8.3; Solarbio, Beijing, China) for 15 min at room temperature. Stained cells were subsequently imaged.

### Lentivirus transduction

Lentiviral vectors were purchased from Genecarer (Xi’an, China). MC3T3-E1 cells were transduced with pLJM1-EGFP (Con; Addgene plasmid, #19319) or ARG2-overexpressing lentiviral vectors (CMV-ARG2) for ARG2 overexpression. The sequences used for overexpression constructs were as follows: mTXN2-overexp-F: GCCACCACGCGTCGACACCATGGCTCAGCGGCT; mTXN2-overexp-R: AGCTGGGTGCTCTAGTCAGCCAATCAGCTTCTTCAGGA. The short hairpin RNA (shRNA) sequences targeting mouse genes were as follows: mShHDAC1-F: TGAAGCCTCACCGAATCCGCAT; mShHDAC1-R: TGGTCATCTCCTCAGCATTGGC; mShHDAC2-F: GTTTTGTCAGCTCTCCACGGGT; mShHDAC2-R: CTTGGCATGATGTAGTCCTCCAG; mShHDAC4-F: AGCAGGAGCTGCTCTTCAGACA; mShHDAC4-R: ACAGAGGTCTGTGGCTGCCAAA; mShHDAC9-F: CTTGAAGGTGCGGTCCAGGTTA; mShHDAC9-R: GCTGCTACTGACCGAGGATTCT. Recombinant adenoviral constructs targeting mouse ARG2 driven by the U6 promoter (rAd/U6-ARG2-shRNA), its corresponding control (rAd/U6-LacZ-shRNA), as well as rAd/CMV empty vector and rAd/CMV-ARG2 were generated in-house. For lentiviral transduction, MC3T3-E1 cells were seeded and cultured until approximately 30% confluence, and then infected with lentiviral particles carrying gene-specific RNAi constructs or non-targeting control vectors for 72 h. After 48 h of infection, puromycin (2 μg/mL) was added to the culture medium to select stably transduced cells. Gene knockdown or overexpression efficiency was confirmed by quantitative PCR (qPCR) and Western blot analysis.

### Luciferase activity assay

Luciferase reporter assays were performed using the dual-luciferase reporter assay system (Promega, Madison, USA) according to the manufacturer’s instructions. Briefly, the wild-type (WT: 5′-GGGATTTTCC-3′) or mutant (MUT: 5′-CCCTAAAAGG-3′) TXN2 promoter region within the p65-binding sequence predicted using the JASPAR database was synthesized and cloned into the pmirGLO dual-luciferase vector (Promega, USA) between the XbaI and SacI restriction sites, generating the pmirGLO-TXN2-WT and pmirGLO-TXN2-MUT reporter constructs, respectively. HEK-293T cells were seeded into 24-well plates and cultured to approximately 70% confluence prior to transfection. Cells were co-transfected with the indicated reporter plasmids together with pLJM1-p65 and/or pLJM1-HDAC9 expression plasmids using Lipo6000™ transfection reagent according to the manufacturer’s protocol. For inhibition experiments, cells were treated with Iohexol as indicated after transfection. After 48 h, cells were lysed using 1× Passive Lysis Buffer (Promega Corporation), and the lysates were centrifuged at 12,000 rpm at 4 °C for 10 min. The supernatants were collected, and firefly and Renilla luciferase activities were sequentially measured using a BioTek microplate reader. Relative luciferase activity was calculated as the ratio of firefly luciferase activity to Renilla luciferase activity.

### Determination of ROS generation

Mitochondrial reactive oxygen species (ROS) production was detected using the MitoSOX fluorescent probe (MedChemExpress, Shanghai, China). Briefly, cells were incubated with 1 μM MitoSOX for 40 min at 37 °C prior to analysis. Fluorescence intensity (Ex/Em = 510/580 nm) was measured using a confocal laser scanning microscope.

### Transmission electron microscope

MC3T3-E1 cells were harvested by digestion and collected for analysis. Cells were fixed with 2.5% glutaraldehyde in PBS (pH 7.4) at 4 °C for 2 h. After washing three times with PBS, samples underwent standard procedures including dehydration, permeabilization, embedding, sectioning, and staining. The ultrastructural morphology of cells was examined using transmission electron microscopy.

### Determination of mitochondrial membrane potential

Mitochondrial membrane potential (ΔΨm) was assessed using the fluorescent dye JC-1 (Solarbio, Beijing, China). Briefly, cells cultured in confocal dishes were incubated with JC-1 working solution (1 μM JC-1 in 500 μL incubation buffer) in the dark at 37 °C for 30 min. Cells were then washed twice with incubation buffer and immediately observed under a confocal laser scanning microscope.

### Co-Immunoprecipitation (Co-IP)

MC3T3-E1 cells (4 × 10^6^ cells per sample) were collected and lysed in ice-cold cell lysis buffer for Western and IP, containing both a protease inhibitor cocktail (APExBIO, Houston, Texas, USA) and a phosphatase inhibitor cocktail (APExBIO, Houston, Texas, USA). Cell lysates (1%) were preserved as inputs. RELA antibody (diluted at 1:50) and Protein A-G Magnetic Beads (Santa Cruz Biotechnology, Shanghai, China) were used to perform immunoprecipitation. Western blot was performed as previously described 24 with primary antibodies and horseradish peroxidase-linked secondary antibody.

### Chromatin immunoprecipitation-quantitative PCR assay

Chromatin immunoprecipitation was performed to examine the recruitment of NF-κB p65 and HDAC9 to the TXN2 promoter. Briefly, MC3T3-E1 cells subjected to the indicated treatments were crosslinked with 1% formaldehyde for 10 min at room temperature, followed by quenching with 125 mM glycine for 5 min. The cells were collected, lysed, and sonicated to generate chromatin fragments predominantly ranging from 200 to 500 bp. Equal amounts of sheared chromatin were incubated overnight at 4°C with antibodies against p65 or HDAC9 or with species-matched normal IgG as a negative control. The immune complexes were captured using protein A/G magnetic beads and sequentially washed with low-salt, high-salt, LiCl, and TE buffers. Chromatin was eluted, and the protein–DNA crosslinks were reversed by overnight incubation at 65°C. Following RNase A and proteinase K digestion, DNA was purified and subjected to quantitative PCR using primers spanning the predicted p65-binding region within the mouse TXN2 promoter. A distal genomic region lacking the predicted p65-binding motif was used as a negative control. Enrichment was calculated using the percent-input method. All experiments were performed independently at least three times. The primer sequences used for ChIP-qPCR were as follows: mTXN2-ChIP-F: AGGCTTCCTGGAGATGACTG; mTXN2-ChIP-R: CCTATTGCATGGCCCCAGCT; mTXN2-ChIP-F; distal negative region-ChIP-F: TTGTCTAGCCCAAACTGACC; distal negative region-ChIP-R: GGAAGCAAGATGCATGGAAA.

### Statistical analysis

All data are presented as the mean ± standard deviation (SD). Comparisons between two groups were performed using a two-tailed Student’s t-test, and comparisons among multiple groups were conducted using one-way analysis of variance (ANOVA) followed by Fisher’s least significant difference post hoc test. All statistical analyses were performed using SPSS version 25.0 (IBM Corp., Armonk, NY, USA). A P value < 0.05 was considered statistically significant.

## Results

### ARG2 is aberrantly upregulated in osteoblasts in mice with senile osteoporosis

D-galactose (D-gal) administration is widely used to model age-related physiological changes, including senile osteoporosis 25, 26. In the present study, qRT-PCR analysis revealed significant upregulation of senescence-associated marker genes (p16, p21, and p53) in the femoral bone tissues of both D-galactose-induced and naturally aged mice (Figure S1A), which was further confirmed by immunoblotting (Figure S1B). Micro-computed tomography (micro-CT) analysis confirmed osteoporotic phenotypes in both models, characterized by trabecular deterioration and reduced bone mass, closely recapitulating age-related bone loss (Figure S1C). Consistently, volumetric bone mineral density (vBMD) and cortical vBMD at the proximal femur were significantly decreased in D-gal-treated and naturally aged mice compared with control animals (Figure S1D). Distal femoral microarchitecture evaluation analyses demonstrated significant reductions in bone volume fraction (BV/TV) and trabecular number (Tb.N), accompanied by increases in structure model index (SMI) and trabecular separation (Tb.Sp) in aged mice (Figure S1E). Cortical bone parameters analysis revealed remarkable decreases in cortical bone volume fraction (Ct.BV/TV) and cortical thickness (Ct.Th) (Figure S1E). Additionally, serum levels of procollagen type I N-terminal propeptide (PINP) and C-terminal telopeptide of type I collagen (CTX-I), markers of bone formation and bone resorption, respectively, were significantly decreased in aged mice, indicating an overall decline in bone remodeling activity (Figure S1F). Histomorphometric analysis using calcein double labeling further exhibited a marked decrease in mineral apposition rate (MAR) in aged mice (Figure S1G). Hematoxylin and eosin (H&E) staining revealed decreased trabecular number and thickness, providing additional evidence of age-associated bone loss (Figure S1H). At the molecular level, qRT-PCR analysis showed that the mRNA expression levels of osteoblast-related genes (Runx2, Bglap, and Alp) were significantly decreased, whereas osteoclast-associated genes (Trap, Nfatc1, and Ctsk) were markedly upregulated in aged mice (Figure S1I-J). Notably, the protein expression level of ARG2 was substantially elevated in femoral bone tissues from both D-gal-treated and naturally aged mice (Figure S1B). To identify the cellular sources of increased ARG2 expression, bone marrow mesenchymal stem cells (BMSCs) and bone marrow mononuclear cells (BMMs) were isolated from mouse femurs and induced to differentiate into osteoblasts and osteoclasts, respectively. Successful lineage differentiation was validated by the increased expression of lineage-specific markers, including Runx2, Alp, and Bglap in osteoblasts, and Trap, Nfatc1, and Ctsk in osteoclasts (Figure S1K-L). Importantly, age-associated upregulation of ARG2 was observed predominantly in osteoblasts rather than osteoclasts (Figure S1M). Thus, analyses across two complementary *in vivo* aging models, together with lineage-specific differentiation experiments, consistently demonstrated that age-associated bone loss is accompanied by enhanced osteoblast senescence, impaired bone formation, and preferential elevation of ARG2 in the osteoblast lineage. These findings provided the rationale for subsequently investigating the functional contribution of osteoblastic ARG2 to age-associated osteoporosis.

### Osteoblast-specific ARG2 knockout ameliorates age-related osteoporosis in mice

To figure out the role of ARG2 in age-related osteoporosis, we generated osteoblast-specific ARG2 conditional knockout (obARG2-cKO) mice by crossing ARG2^flox/flox^ mice with *Osteocalcin-Cre* mice (*Oc-Cre*). Homozygous obARG2-cKO (*Oc-Cre*^+^; ARG2^flox/flox^) mice were screened by PCR genotyping of tail biopsy DNA (Figure 1A). qRT-PCR confirmed a significant decrease of ARG2 expression in femoral bone tissues of obARG2-cKO mice (Figure 1B). To verify osteoblast-specific ARG2 deletion, primary osteoblasts were isolated from ARG2^flox/flox^ mice and obARG2-cKO mice (*Oc-Cre*^+^; ARG2^flox/flox^). qRT-PCR and Western blot analyses confirmed a significant decrease in ARG2 expression at both the mRNA and protein levels in primary osteoblasts from obARG2-cKO mice (Figure S2A-B). Micro-CT analysis revealed substantial bone loss in D-gal-treated wild-type mice as compared to untreated controls. Conversely, osteoblast-specific deletion of ARG2 significantly preserved bone mass and attenuated D-gal-induced bone deterioration (Figure 1C). Consistent with these findings, volumetric bone mineral density (vBMD) was markedly increased in obARG2-cKO mice relative to D-gal-treated wild-type mice (Figure 1D). Quantitative analysis of bone microarchitecture demonstrated that trabecular BV/TV, Tb.N, Ct.BV/TV and Ct.Th were significantly increased, whereas SMI and Tb.Sp were significantly decreased in obARG2-cKO mice (Figure 1D). Moreover, mice with obARG2-cKO exhibited a significant elevation in MAR as compared to the ARG2^flox/flox^ mice (Figure 1E). Histological examination showed increased trabecular bone content, reduced trabecular separation, and a more organized trabecular architecture in D-gal-treated obARG2-cKO mice compared with D-gal-treated ARG2^flox/flox^ littermates (Figure 1F). Consistently, serum levels of the bone turnover markers PINP and CTX-I, which were markedly reduced following D-gal administration, were restored in obARG2-cKO mice, indicating improved bone remodeling activity (Figure 1G). At the molecular level, D-gal treatment significantly downregulated the osteogenic genes Runx2, Alp, and Bglap, while increasing the expression of the osteoclastogenic transcription factor Nfatc1, without significantly affecting Trap or Ctsk expression (Figure 1H). Importantly, these transcriptional alterations were largely reversed by osteoblast-specific ARG2 deletion (Figure 1H). Moreover, D-gal-treated wild-type aged mice exhibited remarkable elevation of the senescence-associated genes p16, p21, and p53, whereas their expression was significantly downregulated in D-gal-treated obARG2-cKO mice (Figure 1I). These findings were further confirmed by immunoblot analysis (Figure 1J). Moreover, protein levels of the osteogenic markers RUNX2 and osteocalcin (OCN), which were substantially decreased in D-gal-induced aged mice, were restored following osteoblastic ARG2 deletion (Figure 1J). These data suggest that osteoblast-specific ARG2 deficiency effectively attenuates age-related bone loss and microarchitectural deterioration.

### ARG2 orchestrates oxidative stress-induced osteoblast senescence

Osteoblast senescence is well recognized as a key driver of age-related osteoporosis 27. To investigate whether ARG2 is involved in regulating osteoblast senescence, we first differentiated the osteoblast precursor cell line MC3T3-E1 into mature OBs, as confirmed by Alizarin red S staining (ARS) (Figure S3A), alkaline phosphatase (ALP) activity assay (Figure S3B), and increased expression of osteoblast marker genes including Runx2, Bglap, and Alp (Figure S3C). To establish an *in vitro* model of oxidative stress-induced senescence, mature OBs were exposed to hydrogen peroxide (H₂O₂, 0-600 μM) for 24 h. Among the tested concentrations, 200 μM H₂O₂ induced the most pronounced senescent phenotype, as evidenced by significantly increased mRNA expression of p21 and p53 (Figure 2A), which was further validated at the protein level by immunoblotting (Figure 2B). Consistently, H₂O₂ exposure markedly increased the proportion of SA-β-gal-positive cells (Figure 2C) and reduced the expression of the proliferation marker PCNA (Figure 2D). CCK-8 assays further demonstrated that treatment with 200 μM H₂O₂ for 24 h resulted in only a moderate reduction (~20%) in cell viability (Figure S4A), indicating that this condition predominantly induces cellular senescence rather than acute cellular cytotoxicity. Notably, ARG2 expression was significantly upregulated in H₂O₂-induced senescent OBs at both the transcript and protein levels (Figure 2E), suggesting a potential role for ARG2 in oxidative stress-mediated osteoblast senescence. To further validate these findings in a primary osteoblast model, bone marrow-derived mesenchymal stem cells (BMSCs) were isolated from murine femurs (Figure S3D). The identity of isolated BMSCs was confirmed by positive CD44 staining and negative CD34 staining (Figure S3E). Following osteogenic induction, the cells exhibited increased mineralized nodule formation detected by ARS staining (Figure S3F), enhanced ALP activity (Figure S3G), and upregulated expression of osteogenic differentiation markers, including Runx2, Bglap, and Alp (Figure S3H). Consistent with the findings in MC3T3-E1 cells, H₂O₂ treatment (200 μM, 24 h) induced a pronounced senescent phenotype in primary BMSC-derived osteoblasts, characterized by increased expression of the senescence markers p21 and p53 at both mRNA and protein levels, elevated ARG2 expression (Figure S5A-B), and an increased proportion of SA-β-gal-positive cells (Figure S5C). These findings independently confirm that oxidative stress-induced osteoblast senescence is consistently associated with ARG2 upregulation across different osteoblast models. To examine whether ARG2 functionally contributes to H₂O₂-induced osteoblast senescence, ARG2 expression was depleted using lentiviral shRNA, with knockdown efficiency validated by qRT-PCR and immunoblotting (Figure 2F-G). ARG2 depletion significantly attenuated the senescent phenotype induced by H₂O₂, as evidenced by a reduced proportion of SA-β-gal-positive cells (Figure 2H), reduced expression of p21 and p53 (Figure 2I), and restoration of PCNA levels (Figure 2J). Conversely, ectopic overexpression of ARG2 in untreated OBs, confirmed by immunoblotting (Figure 2K), was sufficient to trigger cellular senescence, as demonstrated by increased SA-β-gal-positive cells (Figure 2L), upregulated expression of p21 and p53 (Figure 2K), and decreased PCNA levels (Figure 2M). These results indicate that ARG2 is upregulated in response to oxidative stress and functions as a positive modulator of osteoblast senescence.

### ARG2 promotes OBs senescence by impairing mitophagy

It is well recognized that mitophagy is strongly involved in modulating cellular senescence, which prompts us to ask whether ARG2 regulates OBs senescence by impairing mitophagy. As shown in Figure 3A-B, ARG2 overexpression in non-senescent OBs significantly suppressed mitophagy, as indicated by reduced expression of PINK1, Parkin, and LC3-I/II, together with increased p62 accumulation. Similar alterations were observed following H₂O₂ treatment (Figure 3C-D). Importantly, ARG2 knockdown effectively attenuated H₂O₂-induced mitophagy impairment (Figure 3C-D). Consistent with these biochemical findings, transmission electron microscopy (TEM) revealed that H₂O₂ exposure led to the accumulation of intact mitochondria with few morphological features indicative of mitophagic degradation, whereas ARG2 depletion markedly alleviated these changes (Figure 3E).

To further determine whether ARG2-mediated mitophagy impairment results from defective mitophagy initiation or impaired downstream lysosomal degradation, cells were treated with the lysosomal inhibitor bafilomycin A1 (Baf A1), which blocks lysosomal acidification and is widely used to assess mitophagic flux 28. As shown in Figure S6A-B, Baf A1 treatment effectively inhibited lysosomal degradation, as evidenced by the accumulation of both LC3-II and p62 in control and ARG2-overexpressing cells. Notably, even under lysosomal blockade, ARG2 overexpression still resulted in reduced LC3-II accumulation compared with control cells, together with a further increase in p62 accumulation (Figure S6A-B), indicating that ARG2 suppresses mitophagy primarily by inhibiting mitophagy initiation rather than by disrupting downstream lysosomal degradation. These observations suggest that ARG2 may promote OB senescence by impairing mitophagy. To further test this hypothesis, we employed rapamycin (Rapa), a well-established mitophagy inducer 16, 29, 30, to restore ARG2-induced mitophagy inhibition in OBs. As expected, Rapa treatment significantly rescued mitophagy, as demonstrated by increased levels of PINK1, Parkin, and LC3-I/II and reduced p62 accumulation (Figure 3F-G). TEM analysis further confirmed that Rapa reversed the accumulation of intact, non-degraded mitochondria induced by ARG2 overexpression (Figure 3H). Functionally, restoration of mitophagy by Rapa markedly attenuated ARG2-induced upregulation of the senescence markers p21 and p53 (Figure 3I-J) and reduced the proportion of SA-β-gal-positive senescent OBs (Figure 3K). Following the ARG2-overexpression rescue experiments, we further examined whether restoration of mitophagy could similarly alleviate senescence induced directly by oxidative stress. In H₂O₂-treated osteoblasts, rapamycin restored mitophagy-related molecular changes, including increased expression of PINK1, Parkin, and LC3-I/II and reduced p62 accumulation (Figure S7A-C). Importantly, restoration of mitophagy was accompanied by attenuation of the H₂O₂-induced senescent phenotype, as demonstrated by reduced expression of senescence-associated markers and a decreased proportion of SA-β-gal-positive cells (Figure S7D-E). These results independently reproduce the protective effects of mitophagy restoration observed in ARG2-overexpressing cells and further support impaired mitophagy as a functionally relevant event linking oxidative stress and ARG2 to osteoblast senescence.

### ARG2 suppresses mitophagy by promoting mitochondrial oxidative stress in osteoblasts

ARG2 is predominantly localized in mitochondria, where it serves as a major source of ROS, contributing to oxidative stress and the reduction of mitochondrial membrane potential (ΔΨm) 31. To determine whether ARG2 suppresses mitophagy through excessive production of mitochondrial oxidative stress in osteoblasts, we assessed mitochondrial ROS (mtROS) levels and ΔΨm. We found that overexpression of ARG2 in non-senescent OB significantly increased mtROS production (Figure 4A) and reduced ΔΨm (Figure 4B). To determine whether oxidative stress-induced mitochondrial dysfunction was also evident in primary osteoblasts, mitochondrial ROS and mitochondrial membrane potential were examined in BMSC-derived osteoblasts. H₂O₂ treatment significantly increased MitoSOX fluorescence, indicating enhanced mitochondrial superoxide accumulation (Figure S5D). In parallel, JC-1 staining demonstrated a marked reduction in mitochondrial membrane potential following H₂O₂ exposure (Figure S5E). These findings independently confirm that oxidative stress induces mitochondrial redox imbalance and mitochondrial membrane depolarization in primary osteoblasts, supporting the mitochondrial phenotype observed in MC3T3-E1 cells. Importantly, H₂O₂-induced mtROS elevation and ΔΨm reduction were substantially reversed by ARG2 knockdown (Figure 4C-D). To further assess whether ARG2 promotes osteoblast senescence by dampening mitophagy through mitochondrial oxidative stress, we treated cells with the antioxidant N-acetylcysteine (NAC) to suppress ARG2-induced oxidative stress (Figure 4E-F). As expected, NAC treatment alleviated ARG2-induced mitophagy inhibition, as evidenced by increased expression of PINK1, Parkin, and LC3-I/II, and decreased p62 levels (Figure 4G-H). Importantly, NAC also attenuated ARG2-induced upregulation of senescence markers p21 and p53 (Figure 4H), as well as the number of SA-β-gal-positive senescent OBs (Figure 4I). These findings indicate that ARG2 suppresses mitophagy by promoting mitochondrial oxidative stress, thereby contributing to osteoblast senescence.

### ARG2 promoting mitochondrial oxidative stress contributes to OBs senescence through downregulation of TXN2

To further elucidate the molecular mechanism by which ARG2 promotes mitochondrial oxidative stress, we first sought to identify its downstream target genes. We performed an Interactive Venn Diagram analysis incorporating mitochondrial oxidative stress-related genes (MOSRGs), mitophagy-related genes (MRGs), osteoporosis-related genes (OSRGs), ARG2-related genes (ARG2RGs), and senescence-related genes (SRGs). As shown in Figure 5A, four ARG2-targeted candidate genes were identified: cyclin-dependent kinase inhibitor 1a (Cdkn1a), fas cell surface death receptor (FAS), catalase (CAT), and thioredoxin 2 (TXN2). qRT-PCR analysis revealed that ARG2 overexpression in non-senescent osteoblasts downregulated the expression levels of TXN2, but not Cdkn1a, FAS and CAT (Figure 5B), suggesting that ARG2 may induce mitochondrial oxidative stress by repressing TXN2, thereby leading to mitophagy inhibition and cellular senescence. To test this hypothesis, we overexpressed TXN2 in ARG2-overexpressing osteoblasts, as confirmed by qRT-PCR and Western blotting (Figure 5C-D). Remarkably, TXN2 overexpression attenuated ARG2-induced mitochondrial oxidative stress, evidenced by reduced mtROS levels (Figure 5E) and elevation of ΔΨm (Figure 5F) compared to the ARG2-overexpressing group. Moreover, TXN2 overexpression reversed ARG2-induced accumulation of intact mitochondria (Figure 5G), restored the expression of mitophagy markers PINK1, Parkin, and LC3-I/II (Figure 5H-I), and decreased p62 expression (Figure 5I), indicating restored mitophagic flux. Importantly, TXN2 overexpression significantly attenuated ARG2-induced cellular senescence, as demonstrated by decreased expression of the senescence markers p21 and p53 (Figure 5I-J) and a reduced proportion of SA-β-gal-positive cells (Figure 5K). Notably, TXN2 overexpression did not alter ARG2 expression levels (Figure 5I-J), suggesting that TXN2 functions downstream of ARG2 signaling. To determine whether the functional relationship between ARG2 and TXN2 was reproducible in primary osteoblasts, we performed parallel rescue experiments in BMSC-derived osteoblasts. Consistent with the findings in MC3T3-E1 cells, restoration of TXN2 expression markedly attenuated ARG2-induced mitochondrial oxidative stress, as evidenced by decreased mitochondrial ROS accumulation and restoration of mitochondrial membrane potential (Figure S8A-B). TXN2 overexpression also reversed ARG2-induced impairment in mitophagy-associated markers (Figure S8C-D). Importantly, these mitochondrial improvements were accompanied by attenuation of cellular senescence, including reduced expression of senescence-associated markers (Figure S8D-E). In addition, ARS staining and ALP activity assays demonstrated that ARG2 overexpression significantly impaired the osteogenic differentiation capacity of MC3T3-E1 cells, whereas TXN2 overexpression markedly rescued the ARG2-induced suppression of osteogenic differentiation (Figure 5L-M). We next examined whether the ARG2–TXN2 relationship identified experimentally was also associated with human osteoporosis. Analysis of human osteoporosis expression datasets demonstrated significantly higher ARG2 expression and lower TXN2 expression in osteoporosis samples than in non-osteoporotic controls (Figure S9A-C). Moreover, Pearson correlation analyses demonstrated that ARG2 expression was negatively correlated with TXN2 as well as with the mitophagy-associated genes PINK1 and Parkin (Figure S9D-F). Conversely, TXN2 expression was positively correlated with both PINK1 and Parkin (Figure S9G-H). Together, these results indicate that ARG2 induces mitochondrial oxidative stress and impairs mitophagy through repression of TXN2, thereby driving osteoblast senescence and osteogenic dysfunction.

### ARG2 represses TXN2 expression via activation of IκBα

To further elucidate the mechanism by which ARG2 downregulates TXN2 expression, we performed an interactive Venn diagram analysis integrating TXN2-related genes (TXN2RGs), ARG2-related genes (ARG2RGs), mitophagy-related genes (MRGs), mitochondrial oxidative stress-related genes (MOSRGs), and senescence-related genes (SRGs). As shown in Figure 6A, NFKBIA, which encodes IκBα, a member of the IκB family known to inhibit the NF-κB transcription factor, was identified as a potential regulator linking ARG2 and TXN2. Next, we examined the effect of ARG2 on IκBα activation. Immunoblot analysis demonstrated that ARG2 overexpression significantly promoted IκBα activation, as indicated by elevated phosphorylation levels of IκBα (Figure 6B). To further determine whether mtROS functions upstream of ARG2-induced IκBα/NF-κB activation, ARG2-overexpressing osteoblasts were treated with the mitochondria-targeted antioxidant Mito-TEMPO 16. We observed that Mito-TEMPO markedly attenuated ARG2-induced IκBα phosphorylation, suggesting that mtROS accumulation contributes to ARG2-mediated activation of the IκBα/NF-κB signaling (Figure 6C). We next examined the functional involvement of IκBα in ARG2-mediated TXN2 repression, ARG2-overexpressing cells were treated with BAY 11-7082, a pharmacological inhibitor of IκBα phosphorylation. We observed that BAY 11-7082 restored TXN2 expression without altering ARG2 levels (Figure 6D), suggesting that IκBα functions downstream of ARG2. Furthermore, inhibition of IκBα phosphorylation by BAY 11-7082 attenuated ARG2-induced mitochondrial oxidative stress, as evidenced by reduced mtROS levels (Figure 6E) and enhanced ΔΨm (Figure 6F). BAY 11-7082 also reversed ARG2-induced accumulation in intact mitochondria (Figure 6G), decreases in the expression of mitophagy markers LC3-I/II, PINK1, and Parkin (Figure 6D, 6H), and reduction in p62 expression (Figure 6D). Importantly, BAY 11-7082 treatment significantly mitigated ARG2-induced increases in senescence markers p21 and p53 (Figure 6D, 6I) and reduced the number of SA-β-gal-positive cells (Figure 6J). These findings demonstrate that ARG2 represses TXN2 expression through activation of IκBα, which in turn promotes mitochondrial oxidative stress, mitophagy suppression, and osteoblast senescence.

### Activated IκBα negatively modulates TXN2 expression via the p65-HDAC9 interaction

It is well established that phosphorylation of IκBα leads to its degradation, thereby releasing NF-κB dimers and facilitating their nuclear translocation to regulate target gene transcription 32. In non-senescent OBs, we observed that ARG2 overexpression promoted the nuclear translocation of NF-κB p65, whereas this effect was abolished by treatment with the NF-κB inhibitor BAY 11-7082 (Figure 7A). These findings suggest that ARG2-induced IκBα phosphorylation is associated with activation of nuclear p65 signaling, which contributes to TXN2 transcriptional repression. Previous studies have disclosed that NF-κB p65 can act as a transcriptional repressor by interacting with members of the histone deacetylase (HDAC) family, such as HDAC1 and HDAC2 33. To identify potential HDAC family members involved in p65-mediated regulation of TXN2, we performed bioinformatic analyses integrating NF-κB p65 target genes with HDAC family members and identified HDAC1, HDAC2, HDAC4, and HDAC9 as candidate regulators (Figure 7B). qRT-PCR analysis revealed that knockdown of HDAC9, but not HDAC1, HDAC2, or HDAC4, significantly increased TXN2 expression in non-senescent OBs (Figure 7C-F), indicating a potential role for HDAC9 in TXN2 repression. Furthermore, HDAC9 knockdown, as confirmed by Western blot analysis (Figure S10A), markedly attenuated ARG2-induced downregulation of TXN2 mRNA expression (Figure 7G). Next, we performed the PDBePISA interface analysis, which revealed a potential binding interface between NF-κB p65 and HDAC9 (Figure 7H), and this interaction was experimentally validated by co-immunoprecipitation (Co-IP) assays in OBs (Figure 7I). To identify a pharmacological approach for disrupting the p65–HDAC9 interaction, molecular docking analysis was subsequently performed and identified iohexol as a candidate compound capable of interacting with the predicted p65–HDAC9 interface (Figure S10B-C). CCK-8 analysis demonstrated that treatment with iohexol at 25 mg/mL for 24 h maintained acceptable osteoblast viability under the experimental conditions (Figure S10D), and this concentration was therefore selected for subsequent mechanistic experiments. Importantly, Co-IP analysis subsequently demonstrated that iohexol markedly disrupted the ARG2-enhanced interaction between p65 and HDAC9 (Figure 7J), providing experimental support for using iohexol as a tool to interrogate the functional importance of this protein complex.

To further determine whether the p65–HDAC9 complex directly occupies the endogenous TXN2 promoter, we performed ChIP-qPCR assays using primers spanning the predicted p65-binding region. Sequence analysis of the mouse TXN2 promoter identified a putative NF-κB/p65-binding motif (GGGAGGTTCC; JASPAR relative score = 0.8656) (Figure S10E). ARG2 overexpression significantly increased the enrichment of both p65 and HDAC9 at the TXN2 promoter (Figure S10F-G). Iohexol treatment substantially decreased HDAC9 enrichment and reduced p65 occupancy at the TXN2 promoter, consistent with disruption of the p65–HDAC9 complex (Figure S10F-G). No significant enrichment was detected at a distal promoter region lacking the predicted p65-binding motif (Figure S10F-G). These findings provide direct evidence that ARG2-induced NF-κB activation promotes recruitment of the p65–HDAC9 complex to the endogenous TXN2 promoter. Based on the predicted p65-binding motifs within the TXN2 promoter identified using the JASPAR database (Figure 7K), we constructed luciferase reporter plasmids containing either the wild-type TXN2 promoter (TXN2-WT) or a mutant promoter harboring disrupted p65-binding sites (TXN2-MUT). Dual-luciferase reporter assays revealed that p65 overexpression significantly reduced the luciferase activity of the TXN2-WT reporter (Figure 7L). Notably, co-expression of HDAC9 further enhanced this inhibitory effect, indicating cooperative repression of TXN2 transcription by p65 and HDAC9. Importantly, treatment with iohexol, which disrupts the p65-HDAC9 interaction, partially restored the suppressed luciferase activity induced by p65 and HDAC9 co-expression (Figure 7L). In contrast, the TXN2-MUT reporter exhibited significantly higher basal luciferase activity than the TXN2-WT reporter, and neither p65 nor HDAC9 exerted a significant inhibitory effect on the mutant construct (Figure 7L). Consistent with these results, iohexol restored ARG2-suppressed TXN2 expression at both the mRNA and protein levels (Figure 8A, 8G). These findings indicate that the predicted p65-binding region is required for the transcriptional repression of TXN2. Functionally, iohexol treatment mitigated ARG2-induced mitochondrial oxidative stress, as indicated by reduced mitochondrial ROS production (Figure 8B) and restoration of ΔΨm (Figure 8C-D). It also decreased the accumulation of damaged mitochondria induced by ARG2 overexpression (Figure 8E), restored the expression of mitophagy markers PINK1 and Parkin (Figure 8F), normalized LC3-I/II levels, and diminished p62 accumulation (Figure 8G). Furthermore, iohexol attenuated the ARG2-induced upregulation of the senescence markers p21 and p53 (Figure 8G-H) and decreased the proportion of SA-β-gal-positive cells (Figure 8I). These findings indicate that ARG2 promotes IκBα phosphorylation and subsequent nuclear accumulation of p65, which interacts with HDAC9 to suppress TXN2 expression, in turn contributing to mitochondrial oxidative stress, impaired mitophagy, and osteoblast senescence. Furthermore, in primary osteoblasts derived from naturally aged mice, TXN2 overexpression significantly reduced the expression of senescence-associated markers, including p21 and p53, enhanced mitophagy, decreased mitochondrial ROS accumulation, and restored mitochondrial membrane potential (Supplementary Figure S11A-C). Moreover, treatment with the NF-κB inhibitor BAY 11-7082 or the p65–HDAC9 interaction inhibitor iohexol exhibited similar protective effects, further supporting the involvement of the ARG2/IκBα–NF-κB/HDAC9/TXN2 pathway under physiological aging conditions (Supplementary Figure S11A-C). These new findings are consistent with the results obtained from D-galactose- and H₂O₂-induced aging models and provide additional evidence to support our findings in primary osteoblasts under physiological aging conditions.

### BEC alleviates H₂O₂-induced osteoblast senescence *in vitro* and age-associated osteoporosis *in vivo*

The data presented so far begin to paint a picture that targeting ARG2 may be a promising therapeutic target for ameliorating OBs senescence and aging-related osteoporosis. BEC, a widely used pharmacological inhibitor of arginase activity 34, exhibited significant suppression on H₂O₂-induced elevation in arginase activity in MC3T3-E1 cells (Figure S12A). At the signaling level, BEC markedly attenuated H₂O₂-induced IκBα phosphorylation (Figure S12B), restored TXN2 expression at both the protein and transcript levels (Figure S12B-C). These molecular changes were accompanied by reduced mitochondrial ROS accumulation (Figure S12D), and restoration of mitochondrial membrane potential (Figure S12E). Consistent with these improvements in mitochondrial dysfunction, BEC alleviated H₂O₂-induced mitochondrial abnormalities and restored mitophagy-associated markers, including PINK1, Parkin, and LC3-I/II, while reducing p62 accumulation (Figure S12B, F-G). Functionally, BEC restored PCNA expression, reduced the induction of p21 and p53, and significantly decreased the proportion of SA-β-gal-positive osteoblasts following H₂O₂ exposure (Figure S12B, H-J). Thus, pharmacological arginase inhibition recapitulated several key effects of genetic ARG2 depletion, including restoration of TXN2 expression, improvement of mitochondrial homeostasis and mitophagy, and attenuation of osteoblast senescence. To further evaluate the functional importance of TXN2 downstream of ARG2 *in vivo*, we performed a systemic TXN2-overexpression rescue experiment in D-galactose-treated mice using tail-vein administration of TXN2-overexpressing lentivirus. Functionally, TXN2 overexpression significantly ameliorated D-gal-induced bone loss and microarchitectural deterioration, as evidenced by increased trabecular and cortical vBMD, BV/TV, and Tb.N, together with reduced Tb.Sp and SMI (Figure S13A-C). These improvements were accompanied by increased serum PINP levels, partial restoration of CTX-I levels, and preservation of trabecular architecture (Figure S13D-E). TXN2 overexpression also enhanced osteogenic marker expression with minimal effects on osteoclast-related genes and significantly reduced the senescence-associated markers p21 and p53 (Figure S13F-H, J). Successful TXN2 overexpression in vertebral tissues was confirmed at the mRNA and protein levels and was accompanied by restoration of mitophagy-related protein alterations (Figure S13I-J). Furthermore, calcein double labeling demonstrated increased mineral apposition rate (Figure S13K). These findings support a functional role for TXN2 as a downstream target of ARG2 in mitigating age-associated bone deterioration.

To further evaluate the *in vivo* effects of BEC, mice with D-gal-induced osteoporosis were administered BEC for 6 weeks (Figure 9A). Remarkably, BEC administration significantly ameliorated D-gal-induced trabecular micro-architectural deterioration and bone loss. Specifically, the reductions in BV/TV and Tb.N, as well as the increases in SMI and Tb.Sp observed in D-gal-treated mice, were markedly improved following BEC treatment (Figure 9B-C). In addition, BEC significantly restored cortical bone parameters, including cortical vBMD, cortical BV/TV, and Ct.Th, which were reduced in aged mice (Figure 9C). Histological analysis further demonstrated that BEC effectively alleviated the loss and thinning of trabecular bone induced by D-gal treatment (Figure 9D). Furthermore, the decreases in serum bone turnover markers, including PINP and CTX-I, were significantly restored by BEC administration (Figure 9E). Consistent with these findings, BEC improved MAR, indicating enhanced bone formation activity (Figure 9F). At the molecular level, BEC restored the expression of osteoblast-related genes (Runx2, Alp, and Bglap) and reversed the upregulation of osteoclast-associated genes (Trap, Nfatc1, and Ctsk) in aged mice (Figure 9G). Moreover, the mRNA expression of the mitophagy regulators PINK1 and Parkin was significantly reduced in D-gal-treated mice but was restored following BEC administration (Figure 9H). In parallel, BEC markedly suppressed the D-gal-induced upregulation of senescence-associated genes, including p16, p21, and p53 (Figure 9I). Western blot analysis further revealed that BEC reversed the D-gal-induced increases in p21, p53, p62, and phosphorylated IκBα, while restoring the expression of TXN2 and LC3-I/II (Figure 9J). These findings indicate that BEC attenuates oxidative stress, inflammatory signaling, and impaired autophagic flux in aged bone tissue. Next, we determined whether the protective effects of BEC were reproducible in physiological aging. Naturally aged mice exhibited pronounced trabecular and cortical bone deterioration compared with young controls, whereas BEC treatment significantly improved bone mass and microarchitectural parameters, including vBMD, BV/TV, Tb.N, Tb.Sp, SMI, and Ct.Th (Figure S14A-C). These structural improvements were accompanied by partial restoration of serum bone turnover markers, preservation of trabecular morphology, and enhanced expression of osteoblast-associated genes (Figure S14D-G). Moreover, BEC attenuated age-associated cellular senescence and partially restored the ARG2/IκBα/TXN2–mitophagy signaling profile, as reflected by changes in p21, p53, p-IκBα, TXN2, p62, and LC3 expression (Figure S14H-I). Calcein double labeling further demonstrated an increased mineral apposition rate following BEC treatment, indicating improved bone formation (Figure S14J). These findings extend the results obtained in the D-galactose model and demonstrate that arginase inhibition with BEC also attenuates age-associated bone loss in naturally aged mice, providing an important independent validation of the *in vivo* phenotype in a physiological aging context. Furthermore, to determine whether the molecular signaling changes identified in H₂O₂-treated osteoblasts were also present in osteoblasts undergoing physiological aging, primary osteoblasts were isolated from naturally aged mice and age-matched young controls. Osteoblasts derived from aged mice exhibited significantly increased ARG2 expression and enhanced IκBα phosphorylation, accompanied by reduced TXN2 expression (Figure S15A-B). This signaling pattern closely reproduced the ARG2/IκBα/TXN2 alterations observed in the H₂O₂-induced cellular model and in aged bone tissue. These findings therefore provide independent evidence that activation of the ARG2/IκBα axis and suppression of TXN2 are not restricted to an experimentally induced oxidative stress model but are also associated with physiological osteoblast aging. Together with the osteoblast-specific ARG2 genetic model, these findings support an important role for the ARG2–IκBα/NF-κB–TXN2–mitophagy pathway in osteoblast senescence and age-associated bone loss.

## Discussion

Senile osteoporosis, a prevalent age-related skeletal disorder, is characterized by progressive bone loss and microarchitectural deterioration resulting from impaired bone remodeling, particularly osteoblast dysfunction and senescence 35, 36. To investigate the mechanisms underlying osteoblast aging, we employed both H₂O₂- and D-galactose-induced senescence models. H₂O₂ is widely used to induce oxidative stress-mediated cellular senescence, whereas D-galactose promotes a multifactorial aging phenotype that more closely resembles physiological aging by inducing metabolic disturbances and systemic senescence 37-39. In this study, we identified ARG2, a mitochondrial enzyme, as a key contributor to osteoblast senescence during aging. ARG2 was markedly upregulated in aged osteoblasts, where it promoted senescence by suppressing mitophagy through enhanced mitochondrial oxidative stress. This process is mediated by ARG2-dependent repression of TXN2, a mitochondrial antioxidant protein. Specifically, ARG2 increased the phosphorylation of IκBα to facilitate NF-κB p65 nuclear translocation and its interaction with HDAC9, in turn resulting in repression of TXN2 transcription. Importantly, both osteoblast-specific deletion of ARG2 and pharmacological inhibition with BEC effectively attenuated osteoblast senescence and maintained bone mass in D-galactose-induced and naturally aged mouse models. These findings disclose a previously unrecognized ARG2–TXN2–mitophagy signaling axis that contributes to osteoblast senescence and age-related bone loss and suggest ARG2 as a potential therapeutic target for age-related bone loss.

Our findings expand the current understanding of ARG2 in senile osteoporosis and establish its previously unrecognized role in aging pathologies beyond vascular and immune cells. ARG2 has been implicated in endothelial and vascular smooth muscle cell senescence, where it triggers mitochondrial ROS production and disrupts redox homeostasis, thereby leading to vascular aging and atherosclerosis 12, 13. In aging macrophages, ARG2 upregulation has been reported to induce non-cell-autonomous effects on cardiomyocytes and fibroblasts through IL-1β secretion, accelerating cardiac and skeletal muscle aging 40. In osteoarthritis, ARG2 drives cartilage degradation by enhancing matrix metalloproteinase expression in fibroblasts, underscoring its pro-inflammatory and catabolic functions 41. Despite these observations, the role of ARG2 in bone-forming osteoblasts has remained largely unknown. In the present study, ARG2 expression was significantly elevated in osteoblasts from both D-galactose-treated and naturally aged mice and was accompanied by increased expression of the senescence markers p16, p21, and p53, as well as pronounced trabecular bone deterioration. Osteoblast-specific ARG2 knockout mice exhibited preserved bone volume/tissue volume, trabecular number, and serum bone turnover markers, together with improved serum bone turnover markers. Importantly, osteoblast-specific ARG2 deficiency had minimal effects on osteoclast-associated parameters, indicating that the skeletal protection conferred by ARG2 deletion is primarily mediated through osteoblast-autonomous mechanisms. Interestingly, although osteoclast-related genes, particularly Nfatc1, were transcriptionally elevated in aged bone tissues, serum CTX-I levels were reduced. These findings are consistent with a relatively low-turnover bone remodeling phenotype in our aging models. One possible explanation is that the increased expression of osteoclastogenic genes indicates a compensatory or early adaptive transcriptional response to skeletal aging that does not necessarily translate into enhanced osteoclast differentiation or bone-resorptive activity. Therefore, impaired osteoblast function is likely to be the predominant contributor to the bone loss observed in the present model. Nevertheless, because direct histomorphometric analyses of osteoclasts, such as TRAP staining or quantification of osteoclast number or surface, were not performed in this study, this interpretation should be considered with caution and warrants further investigation.

Notably, our study demonstrates that ARG2-mediated osteoblast senescence is closely associated with impaired mitophagy driven by excessive mitochondrial oxidative stress. Defective mitophagy leads to the accumulation of dysfunctional mitochondria, which further exacerbates oxidative stress and promotes the senescence-associated secretory phenotype (SASP), ultimately impairing osteoblast differentiation and bone formation 42, 43. In our H₂O₂-induced osteoblast senescence, ARG2 overexpression reduced the expression of PINK1, Parkin, and LC3-II while increasing p62 levels, indicating impaired mitophagic activity. Transmission electron microscopy further revealed the accumulation of damaged mitochondria in ARG2-overexpressing cells, which was accompanied by increased SA-β-gal staining and reduced proliferative capacity. These findings align with previous studies identifying mitophagy impairment as an early event in cellular senescence. In osteoblasts, advanced glycation end-products (AGEs) suppress SIRT3-PINK1 signaling, which results in defective mitophagy and mitochondrial dysfunction, in turn accelerating cellular senescence 44. Similarly, in the cardiovascular system, ARG2 has been found to impair autophagy to contribute advanced atherosclerosis through regulation of MTOR and PRKAA/AMPK signaling 14. Our data extend these observations to osteoblasts and suggest that ARG2 promotes excessive mtROS generation, resulting in impaired mitophagic flux and disruption of mitochondrial quality control. Given the critical role of mitophagy in maintaining osteoblast viability, function, and differentiation 45, 46, ARG2-mediated suppression of mitophagy may provide an important mechanism underlying age-related skeletal degeneration and osteoporosis. Although mtROS accumulation, loss of mitochondrial membrane potential, ultrastructural alterations, and changes in mitophagy markers consistently demonstrated mitochondrial dysfunction in the present study, direct assessments of mitochondrial bioenergetics, such as ATP production or oxygen consumption rate, were not performed. Future studies incorporating these functional measurements will further characterize the impact of the ARG2–mtROS–mitophagy axis on mitochondrial respiration and energy metabolism in osteoblasts.

Importantly, we identify TXN2, a critical mitochondrial antioxidant enzyme 47, as a direct downstream target negatively regulated by ARG2. ARG2-mediated suppression of TXN2 resulted in elevated mitochondrial oxidative stress, impaired mitophagy, and accelerated osteoblast senescence, whereas TXN2 overexpression effectively reversed these defects. These findings establish TXN2 as a critical mediator of ARG2-induced osteoblast senescence. Although TXN2 haploinsufficiency has been reported to impair mitochondrial function and increase oxidative stress in multiple tissues 48, its regulatory role in osteoblasts has remained largely unexplored. A major mechanistic finding of the present study is the identification of an ARG2–IκBα/NF-κB–HDAC9–TXN2 signaling axis. Specifically, ARG2 promoted IκBα phosphorylation, thereby facilitating p65 nuclear translocation and recruitment of HDAC9 to the TXN2 promoter, ultimately repressing TXN2 transcription. While phosphorylation of IκBα is classically associated with the release of NF-κB from inhibitory sequestration and activation of pro-inflammatory gene expression, our findings reveal a distinct repressive function mediated by p65-HDAC9 complexes. This observation aligns with the established context-dependent roles of NF-κB as both a transcriptional activator and repressor, mediated in part through interactions with histone deacetylases. Among the HDAC family members examined, HDAC9 was identified as the principal mediator of TXN2 suppression. Consistent with its established role in vascular inflammation and atherosclerosis via IKK/NF-κB pathway activation 49, 50, HDAC9 was functionally required for ARG2-induced TXN2 downregulation, as supported by HDAC9 silencing, co-immunoprecipitation, promoter-reporter assays, and structural interaction analyses. Our additional ChIP–qPCR experiments further strengthened this mechanism by demonstrating that ARG2 overexpression increased the occupancy of both p65 and HDAC9 at the endogenous TXN2 promoter, whereas inhibition of IκBα phosphorylation or disruption of the p65–HDAC9 interaction reduced their promoter recruitment. Together with the HDAC9-knockdown and promoter-reporter results, these findings provide direct evidence that HDAC9 functions as an essential component of the p65-associated transcriptional repressor complex responsible for efficient suppression of TXN2 transcription. Consistently, pharmacological disruption of the p65-HDAC9 interaction using iohexol effectively restored TXN2 expression and mitophagic activity, phenocopying the effects of ARG2 inhibition by BEC. These observations highlight the p65–HDAC9 interaction as a mechanistically important regulatory node within the ARG2/TXN2 pathway. Nevertheless, the precise biochemical contribution of HDAC9 to TXN2 repression remains to be fully defined. Because iohexol disrupts the p65–HDAC9 interaction rather than selectively inhibiting HDAC9 enzymatic activity, the present experiments cannot determine whether transcriptional repression requires the deacetylase activity of HDAC9 or instead indicates a non-enzymatic function, such as stabilization or recruitment of the repressor complex. Future studies using validated HDAC9 catalytic mutants or highly selective HDAC9 inhibitors will therefore be necessary to distinguish between catalytic activity-dependent and activity-independent mechanisms. Another unresolved question concerns how mitochondrially localized ARG2 activates the cytoplasmic IκBα/NF-κB pathway. One plausible mechanism involves ARG2-induced mtROS, which may diffuse into the cytoplasm or initiate secondary redox signaling cascades that activate the IKK complex. Indeed, mtROS have been widely reported to oxidize critical cysteine residues in upstream signaling molecules or directly promote IKK activation 51-54, thereby leading to IκBα phosphorylation and NF-κB p65 nuclear translocation. Consistent with this model, ARG2 overexpression markedly increased mtROS production, whereas ARG2 depletion or treatment with either the general antioxidant NAC or the mitochondria-targeted antioxidant MitoTEMPO attenuated IκBα phosphorylation and downstream NF-κB activation. These findings support the concept that excessive mtROS acts as an important retrograde signal linking mitochondrial ARG2 activity to cytoplasmic NF-κB signaling. However, the upstream mechanism by which ARG2 promotes mtROS generation in osteoblasts remains incompletely understood. Further studies will be required to determine whether this process depends on altered mitochondrial substrate metabolism, electron transport chain dysfunction, redox enzyme activity, or other ARG2-dependent mitochondrial processes.

Nevertheless, several limitations should be acknowledged in this study. First, although systemic TXN2 overexpression through tail-vein lentiviral administration provided important evidence supporting the protective role of TXN2 in age-associated osteoporosis, this approach does not exclusively target osteoblasts. The protective effects observed in bone may involve both osteoblast-autonomous mechanisms and potential systemic contributions. Therefore, potential contributions from TXN2 modulation in other tissues cannot be fully excluded. Future studies using osteoblast-specific TXN2 gain-of-function models, e.g., osteoblast-targeted TXN2 transgenic mice or Cre-dependent TXN2 overexpression systems, will be necessary to definitively determine the osteoblast-specific role of TXN2 in age-associated osteoporosis. Second, although primary osteoblasts isolated from naturally aged mice exhibited activation of the ARG2/IκBα–NF-κB/TXN2 signaling axis and TXN2 restoration exerted protective effects in primary osteoblast models, the detailed mechanistic studies, including TXN2 promoter regulation and p65–HDAC9 interaction analyses, were primarily performed in H₂O₂-induced senescent osteoblasts. Future studies using osteoblast-specific genetic models and primary osteoblasts derived from naturally aged animals will further validate the functional contribution of the ARG2–IκBα/NF-κB–HDAC9–TXN2 axis under physiological aging conditions and strengthen the translational relevance of this mechanism. Finally, an additional limitation concerns the pharmacological specificity of BEC. BEC is a competitive arginase inhibitor that inhibits both ARG1 and ARG2 and therefore cannot be considered an ARG2-selective pharmacological agent. Although the effects of BEC were consistent with those observed following osteoblast-specific ARG2 deletion, possible contributions resulting from ARG1 inhibition cannot be excluded, particularly following systemic administration *in vivo*. The BEC experiments should therefore be interpreted as complementary evidence demonstrating the effects of pharmacological arginase inhibition rather than as definitive pharmacological proof of ARG2-specific inhibition. Importantly, the osteoblast-specific ARG2 conditional knockout experiments provide independent genetic evidence supporting the role of ARG2 in osteoblast senescence and age-associated bone loss. However, a well-validated and widely available ARG2-specific inhibitor suitable for *in vivo* studies is currently lacking due to the substantial structural similarity between the catalytic sites of ARG1 and ARG2. Future development of highly selective ARG2 inhibitors will be required to determine whether selective pharmacological targeting of ARG2 can reproduce the skeletal protective effects observed with genetic ARG2 deletion in age-associated osteoporosis.

## Conclusion

In summary, ARG2 acts as a key driver of osteoblast senescence by linking mitochondrial ROS accumulation to impaired mitophagy through downregulation of TXN2. Targeting ARG2 may provide a promising strategy to improve age-associated osteoporosis.

## Supplementary Material

Supplementary figures.

## Figures and Tables

**Figure 1:**
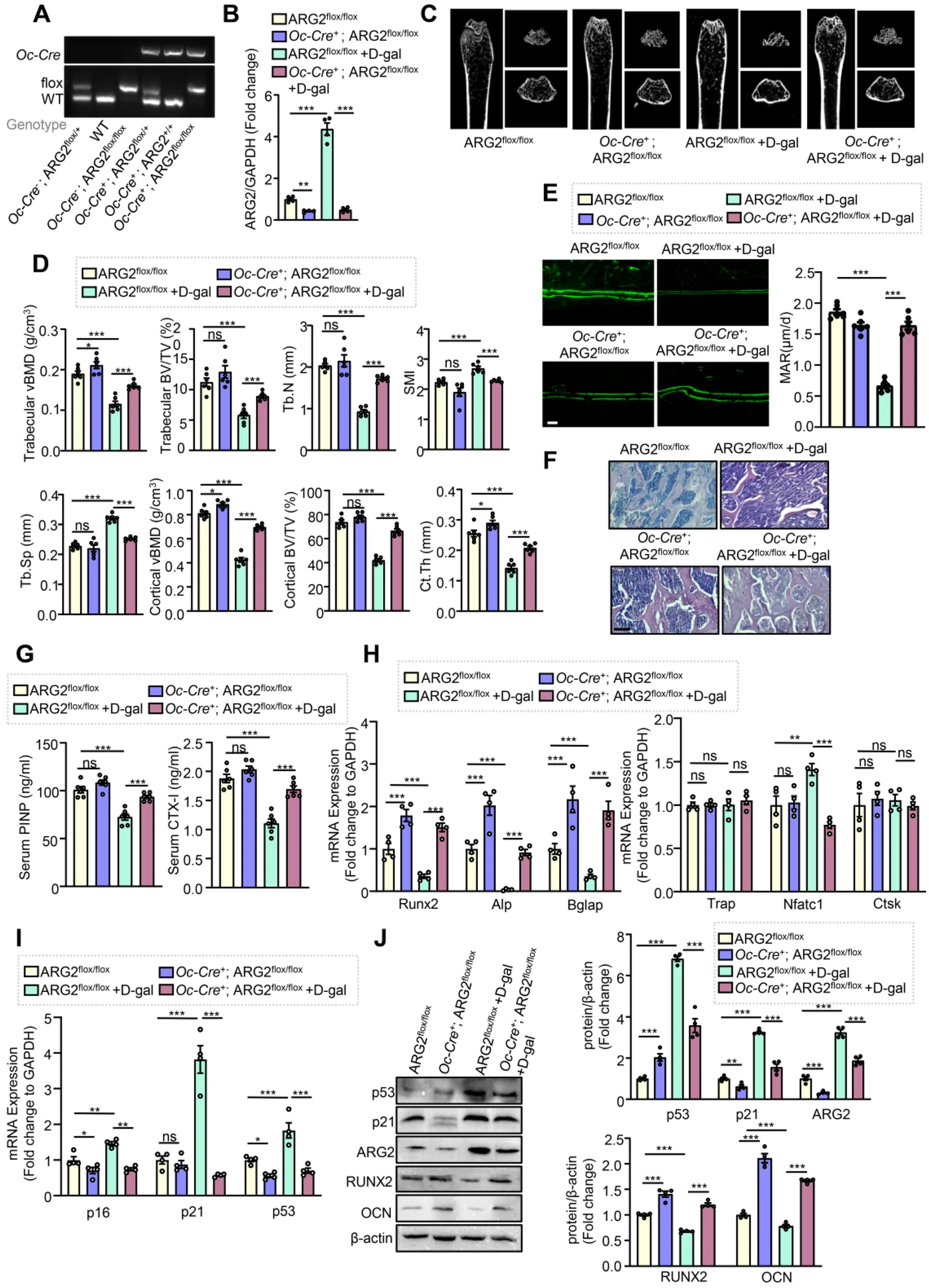
** Osteoblast-specific knockdown of ARG2 alleviates age-related osteoporosis. (A)** Agarose gel electrophoresis of PCR genotyping of genomic DNA from different mouse genotypes. **(B)** Quantification of ARG2 mRNA expression by qRT-PCR. **(C)** Representative images derived from micro-CT analysis of ARG2^flox/flox^ mice, *Oc-Cre^+^* ARG2^flox/flox^ mice, ARG2^flox/flox^ mice with D-galactose and *Oc-Cre^+^* ARG2^flox/flox^ mice with D-galactose (n = 6 per group). **(D)** Quantitative analysis of bone parameters, including trabecular and cortical volumetric bone mineral density (vBMD), trabecular bone volume fraction (BV/TV), cortical BV/TV, structure model index (SMI), trabecular number (Tb.N), trabecular separation (Tb.Sp), and cortical thickness (Ct.Th), based on micro-CT data (n = 6 per group). **(E)** Representative images of calcein double labeling of trabecular bones and quantification of mineral apposition rate (MAR). Scale bar = 50 μm. **(F)** Representative images of HE-stained decalcified vertebral sections from ARG2^flox/flox^ mice, *Oc-Cre^+^* ARG2^flox/flox^ mice, ARG2^flox/flox^ mice treated with D-galactose and *Oc-Cre^+^* ARG2^flox/flox^ mice treated with D-galactose. Scale bar = 500 μm (n = 6 per group). **(G)** Serum levels of bone turnover markers PINP and CTX-I were measured by ELISA (n = 6 per group). **(H)** qRT-PCR analysis of mRNA expression of osteoblast- and osteoclast-related differentiation markers in vertebral bone (n = 4 per group). **(I)** qRT-PCR analysis of senescence-associated gene expression in vertebral bone (n = 4 per group). **(J)** Western blot analysis of p21, p53, RUNX2, OCN, and ARG2 protein expression. Quantification of protein levels is shown in the corresponding bar graphs (right panels). Statistical significance was assessed by one-way ANOVA. Data are presented as mean ± SEM. ns, not significant; *p < 0.05, **p < 0.01, ***p < 0.001 between the indicated groups.

**Figure 2:**
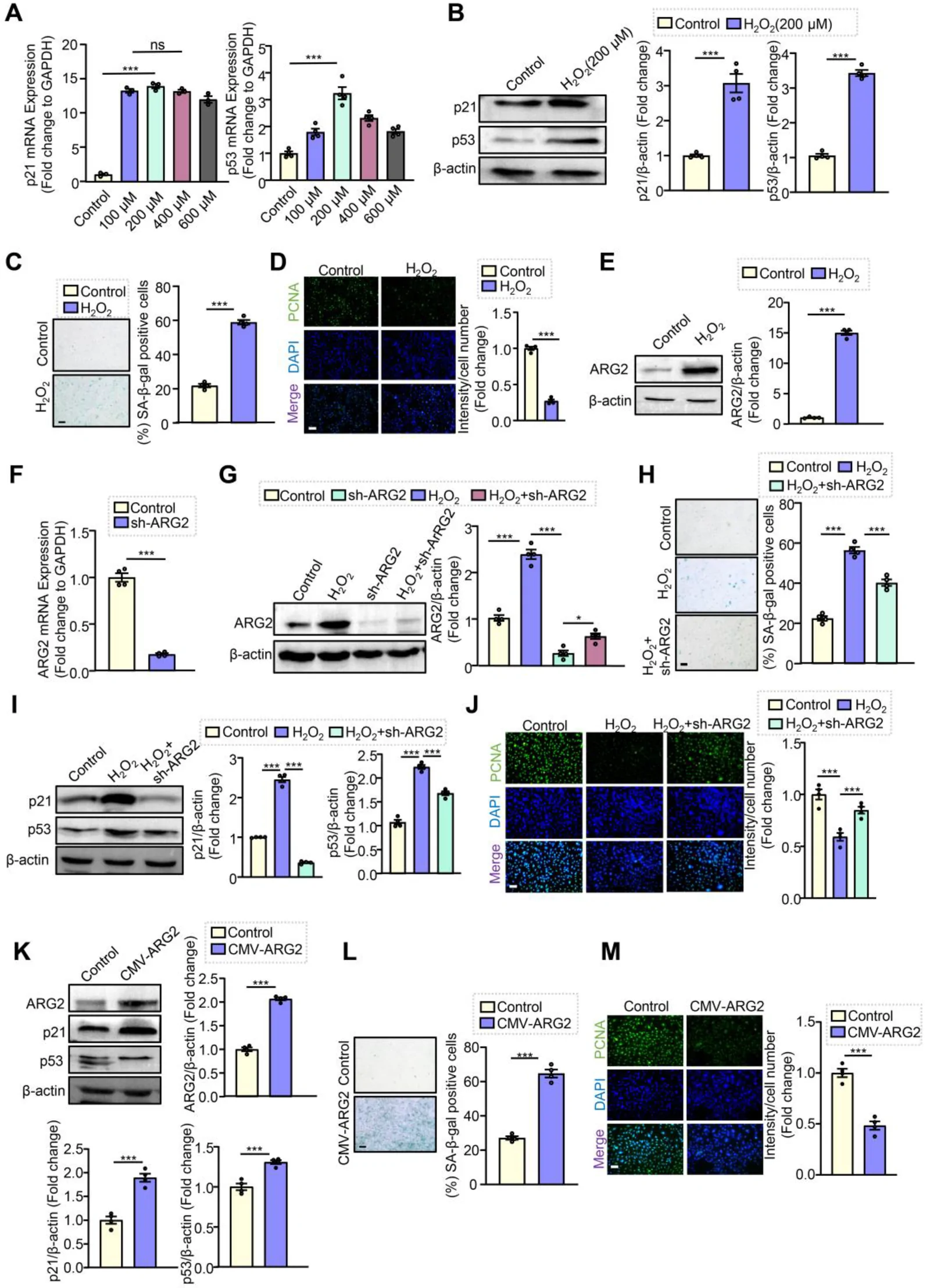
** ARG2 modulates hydrogen peroxide-induced osteoblast senescence. (A)** qRT-PCR analysis of p21 and p53 mRNA levels in MC3T3-E1 cells treated with increasing concentrations of H₂O₂ (0, 100, 200, 400, and 600 μM). Based on these results, MC3T3-E1 cells were subsequently treated with H₂O₂ (200 μM) for 24 h. **(B)** Immunoblot analysis of p21 and p53 protein levels, with corresponding quantification shown on the right. **(C)** SA-β-gal staining and quantification of SA-β-gal-positive cells. **(D)** Representative images and quantification of PCNA immunostaining. **(E)** Immunoblot analysis of ARG2 protein expression, with corresponding quantification shown on the right. MC3T3-E1 cells were transduced with pLKO.1-control (Con) or pLKO.1-ARG2 shRNA (sh-ARG2) lentivirus. **(F)** qRT-PCR validation of ARG2 knockdown efficiency. **(G)** Immunoblot confirmation of ARG2 silencing in H₂O₂-treated osteoblasts. **(H)** SA-β-gal staining and quantification of SA-β-gal-positive cells following ARG2 knockdown. **(I)** Immunoblot analysis of p21 and p53 protein expression after ARG2 depletion, with corresponding quantification shown on the right. **(J)** Representative images and quantification of PCNA immunostaining following ARG2 knockdown. MC3T3-E1 cells were transduced with pLJM1-EGFP (Con) or pLJM1-HA-ARG2 (CMV-ARG2) for ARG2 overexpression. **(K)** Immunoblot analysis of ARG2, p21, and p53 protein levels in ARG2-overexpressing osteoblasts, with corresponding quantification shown on the right. **(L)** SA-β-gal staining and quantification of SA-β-gal-positive cells in ARG2-overexpressing osteoblasts. **(M)** Representative images and quantification of PCNA immunostaining following ARG2 overexpression. Data are presented as mean ± SEM (n = 4). Statistical significance was determined by one-way ANOVA. ns, not significant; ***P < 0.001 versus the indicated groups. Scale bar = 100 μm.

**Figure 3:**
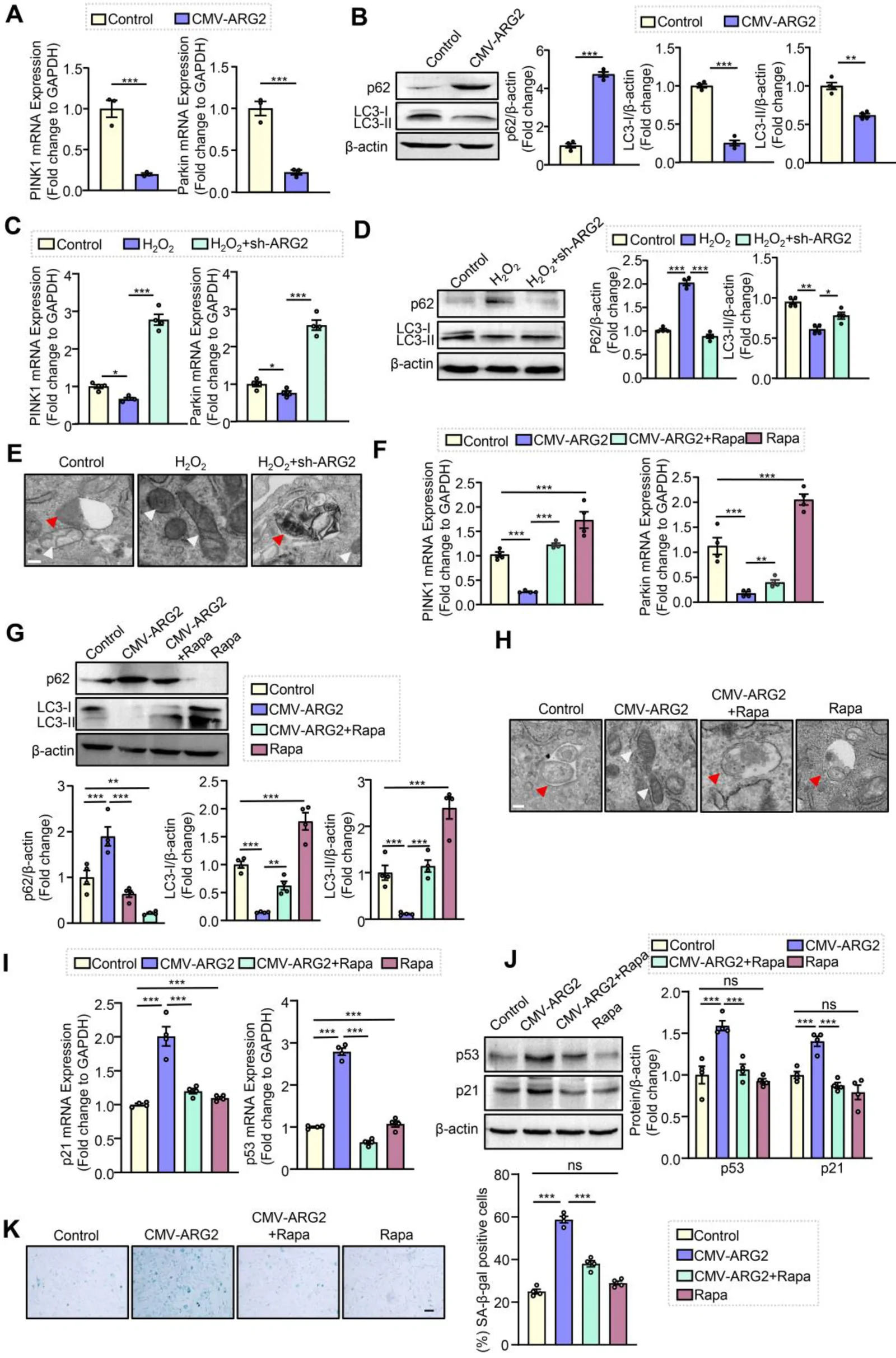
** ARG2 suppresses mitophagy and restoration of mitophagy rescues ARG2-induced cellular senescence in osteoblasts.** MC3T3-E1 cells were transduced with pLJM1-EGFP (Con) or pLJM1-HA-ARG2 (CMV-ARG2) for ARG2 overexpression. **(A)** qRT-PCR analysis of PINK1 and Parkin mRNA expression. **(B)** Immunoblotting analysis of p62 and LC3 protein levels, with corresponding quantification shown on the right. MC3T3-E1 cells were transduced with pLKO.1-control (Con) or pLKO.1-ARG2 shRNA (sh-ARG2) lentivirus for 48 h, followed by treatment with H₂O₂ (200 μM) for 24 h. **(C)** qRT-PCR analysis of PINK1 and Parkin mRNA expression. **(D)** Immunoblotting analysis of p62 and LC3 protein levels, with corresponding quantification shown on the right. **(E)** Representative transmission electron microscopy (TEM) images showing mitophagy. Red arrows indicate mitophagic vesicles, and white arrows indicate mitochondria. Scale bar = 500 nm. MC3T3-E1 cells were transduced with pLJM1-EGFP (Con) or pLJM1-HA-ARG2 (CMV-ARG2) and subsequently treated with rapamycin (Rapa, 100 nM) for 24 h. **(F)** qRT-PCR analysis of PINK1 and Parkin mRNA expression. **(G)** Immunoblotting analysis of p62 and LC3 protein levels, with corresponding quantification shown on the right. **(H)** Representative TEM images showing mitophagy. Red arrows indicate mitophagic vesicles, and white arrows indicate mitochondria. Scale bar = 500 nm. **(I)** qRT-PCR analysis of p21 and p53 mRNA expression. **(J)** Immunoblotting analysis of p21 and p53 protein levels, with corresponding quantification shown on the right. **(K)** Representative senescence-associated β-galactosidase (SA-β-gal) staining images, with quantification of SA-β-gal-positive cells shown on the right. Scale bar = 100 μm. Statistical significance was determined by one-way ANOVA. Data are presented as mean ± SEM (n = 4). ns, not significant; **p < 0.01; ***p < 0.001 versus the indicated groups between the indicated groups.

**Figure 4:**
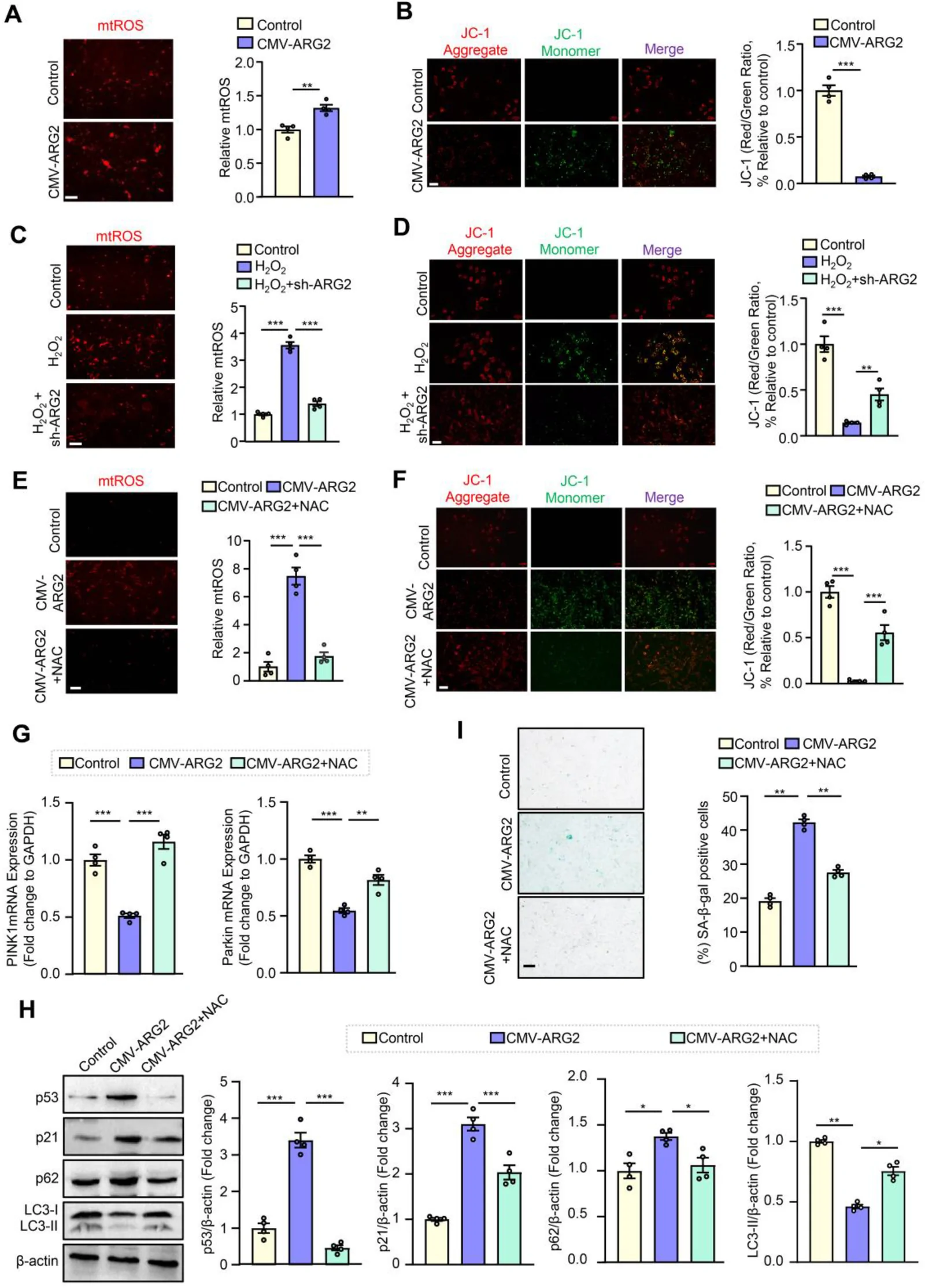
** NAC alleviates ARG2-induced mitophagy suppression and cellular senescence in osteoblasts.** MC3T3-E1 cells were transduced with pLJM1-EGFP control vector (Con) or pLJM1-HA-ARG2 (CMV-ARG2) to induce ARG2 overexpression. **(A)** Mitochondrial superoxide levels were assessed by MitoSOX staining (1 μM). **(B)** Mitochondrial membrane potential (Δψm) was evaluated by JC-1 staining. MC3T3-E1 cells were transduced with pLKO.1-control (Con) or pLKO.1-ARG2 shRNA (sh-ARG2) lentivirus for 48 h, followed by treatment with H₂O₂ (200 μM) for 24 h. **(C)** Mitochondrial superoxide levels were determined by MitoSOX staining (1 μM). **(D)** Δψm was assessed by JC-1 staining. For rescue experiments, MC3T3-E1 cells transduced with pLJM1-EGFP (Con) or pLJM1-HA-ARG2 (CMV-ARG2) were treated with N-acetylcysteine (NAC, 500 μM) for 24 h. **(E)** Mitochondrial superoxide levels were evaluated by MitoSOX staining (1 μM). **(F)** Δψm was assessed by JC-1 staining. **(G)** qRT-PCR analysis of PINK1 and Parkin mRNA expression. **(H)** Immunoblot analysis of p53, p21, p62, and LC3 protein expression. Quantitative analyses of protein levels are shown in the bar graphs on the right. **(I)** Senescence-associated β-galactosidase (SA-β-gal) staining. Bar graphs represent the quantification of SA-β-gal-positive cells. Statistical significance was determined by one-way ANOVA. Data are presented as mean ± SEM (n = 4). Scale bar = 100 μm. *p < 0.05, **p < 0.01, ***p < 0.001 between the indicated groups.

**Figure 5:**
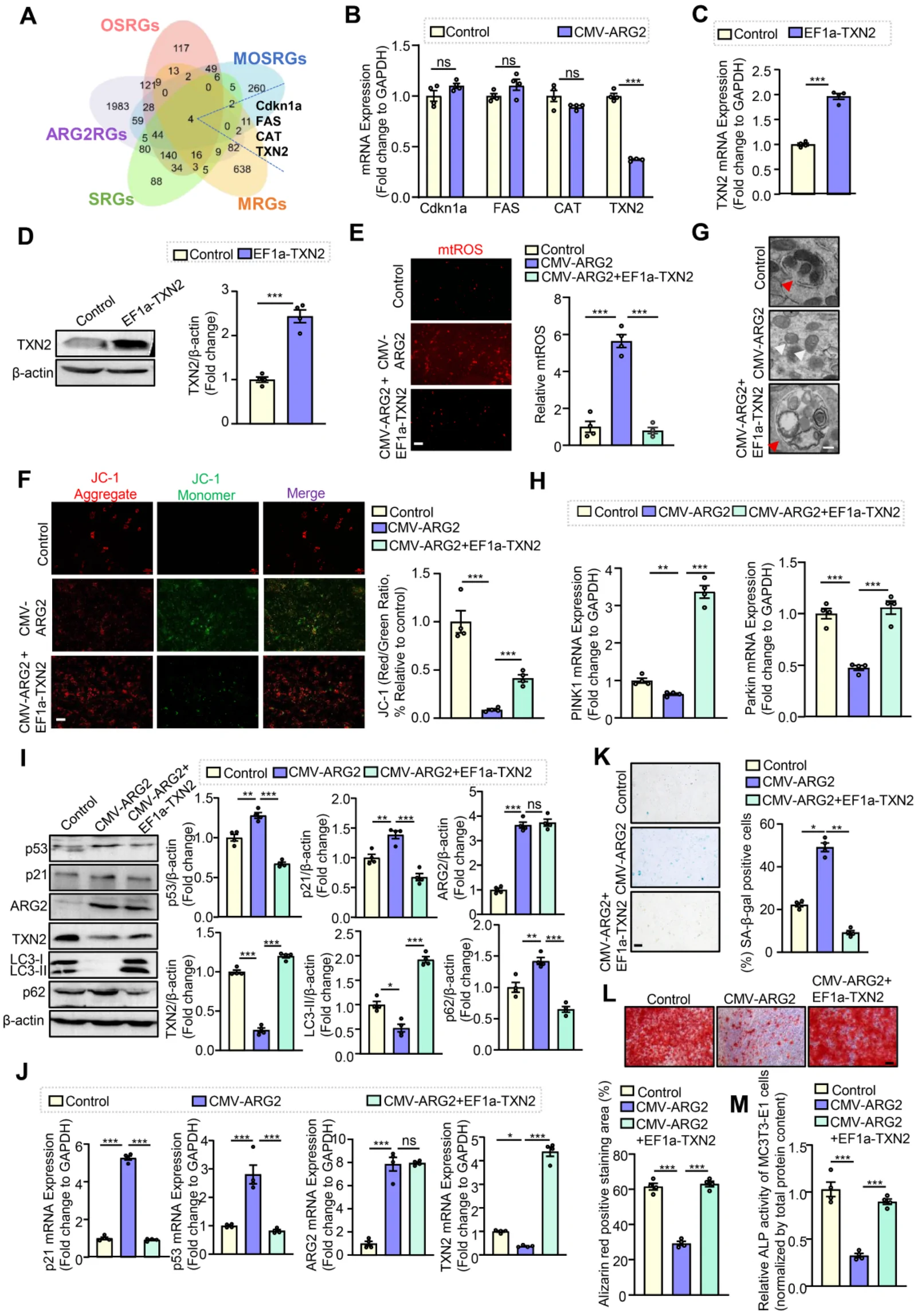
** Overexpression of TXN2 rescues ARG2-induced mitophagy impairment and cellular senescence in osteoblasts. (A)** Venn diagram analysis of mitochondrial oxidative stress-related genes (MOSRGs), mitophagy-related genes (MRGs), osteoporosis-related genes (OSRGs), ARG2-related genes (ARG2RGs), and senescence-related genes (SRGs) retrieved from the GeneCards database. MC3T3-E1 cells were transduced with pLJM1-EGFP (Con) or pLJM1-HA-ARG2 (CMV-ARG2) for ARG2 overexpression. **(B)** qRT-PCR analysis of the mRNA expression of Cdkn1a, FAS, CAT and TXN2. Senescent MC3T3 E1s were transduced with pLJM1-EGFP as a control (Con) or pLJM1-HA-TXN2 (EF1a-TXN2) to overexpress TXN2. **(C)** qRT-PCR analysis of mRNA levels of TXN2 overexpression efficiency in OBs. **(D)** Immunoblotting analysis of TXN2 overexpression efficiency in OBs. The bar graphs on the right show quantifications of protein levels. MC3T3-E1 cells were transduced with pLJM1-EGFP (Con) or pLJM1-HA-ARG2 (CMV-ARG2) for ARG2 overexpression, and subsequently with pLJM1-HA-TXN2 (EF1a-TXN2) to overexpress TXN2. **(E)** Mitochondrial superoxide was stained with 1 μM MitoSOX. Scale bar = 100 μm. **(F)** Δψm Assessment by JC-1 staining. Scale bar = 100 μm. **(G)** Representative TEM images showing mitophagy. Red arrows: mitophagy; White arrows: mitochondria. Scale bar=500 nm. **(H)** qRT-PCR analysis of the mRNA expression of PINK1 and Parkin. **(I)** Immunoblotting analysis of p21, p53, ARG2, TXN2, p62 and LC3 protein expression. The bar graphs on the right show quantifications of protein levels. **(J)** qRT-PCR analysis of the mRNA expression of p21, p53, ARG2 and TXN2. **(K)** SA-β-gal staining. Bar graphs represent quantifications of SA-β-gal-positive cells. **(L)**Alizarin Red S staining (ARS) and quantitative analysis of mineral deposition. Scale bar = 100 μm. **(M)**Alkaline phosphatase (ALP) activity assay. Statistical differences were examined by one-way ANOVA. Data are presented as mean ± SEM. n = 4. ns: not significant, *p < 0.05, **p < 0.01, ***p < 0.001 between the indicated groups.

**Figure 6:**
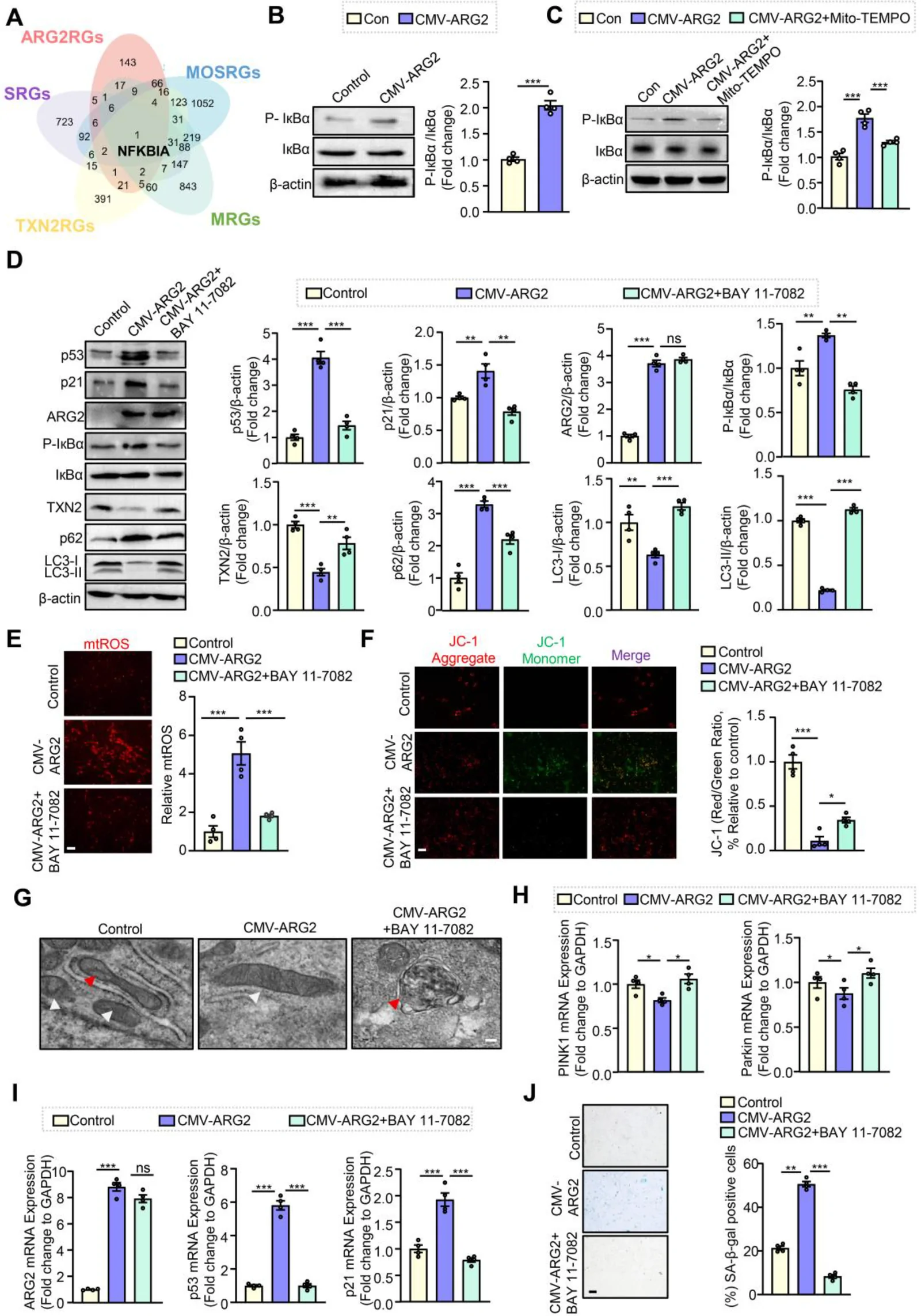
** Inhibition of IκBα phosphorylation reverses ARG2-induced downregulation of TXN2 expression, mitophagy and cellular senescence. (A)** Venn diagram analysis of TXN2-related genes (TXN2RGs), ARG2-related genes (ARG2RGs), mitophagy-related genes (MRGs), mitochondrial oxidative stress-related genes (MOSRGs), and senescence-related genes (SRGs) retrieved from the GeneCards database. MC3T3-E1 cells were transduced with pLJM1-EGFP (Con) or pLJM1-HA-ARG2 (CMV-ARG2) for ARG2 overexpression. **(B)** Immunoblotting analysis of IκBα and P-IκBα protein expression. The bar graphs on the right show quantifications of protein levels. MC3T3-E1 cells were transduced with pLJM1-EGFP (Con) or pLJM1-HA-ARG2 (CMV-ARG2) to induce ARG2 overexpression, followed by treatment with Mito-TEMPO (50 μM) or BAY 11-7082 (10 nM) for 24 h. **(C)** Immunoblotting analysis of IκBα and P-IκBα protein expression. **(D)** Immunoblotting analysis of p21, p53, ARG2, IκBα, P-IκBα, TXN2, p62 and LC3 protein expression. **(E)** Mitochondrial superoxide was stained with 1 μM MitoSOX. Scale bar = 100 μm. **(F)** Δψm assessment by JC-1 staining. Scale bar = 100 μm. **(G)** Representative transmission electron microscopy (TEM) images showing mitophagy. Red arrows indicate mitophagosomes, and white arrows indicate mitochondria. Scale bar = 500 nm. **(H)** qRT-PCR analysis of PINK1 and Parkin mRNA expression. **(I)** qRT-PCR analysis of ARG2, p21, and p53 mRNA expression. **(J)** Senescence-associated β-galactosidase (SA-β-gal) staining. Quantification of SA-β-gal-positive cells is shown in the accompanying bar graphs. Statistical significance was determined by one-way ANOVA. Data are presented as mean ± SEM (n = 4). *p < 0.05, **p < 0.01, ***p < 0.001 between the indicated groups.

**Figure 7:**
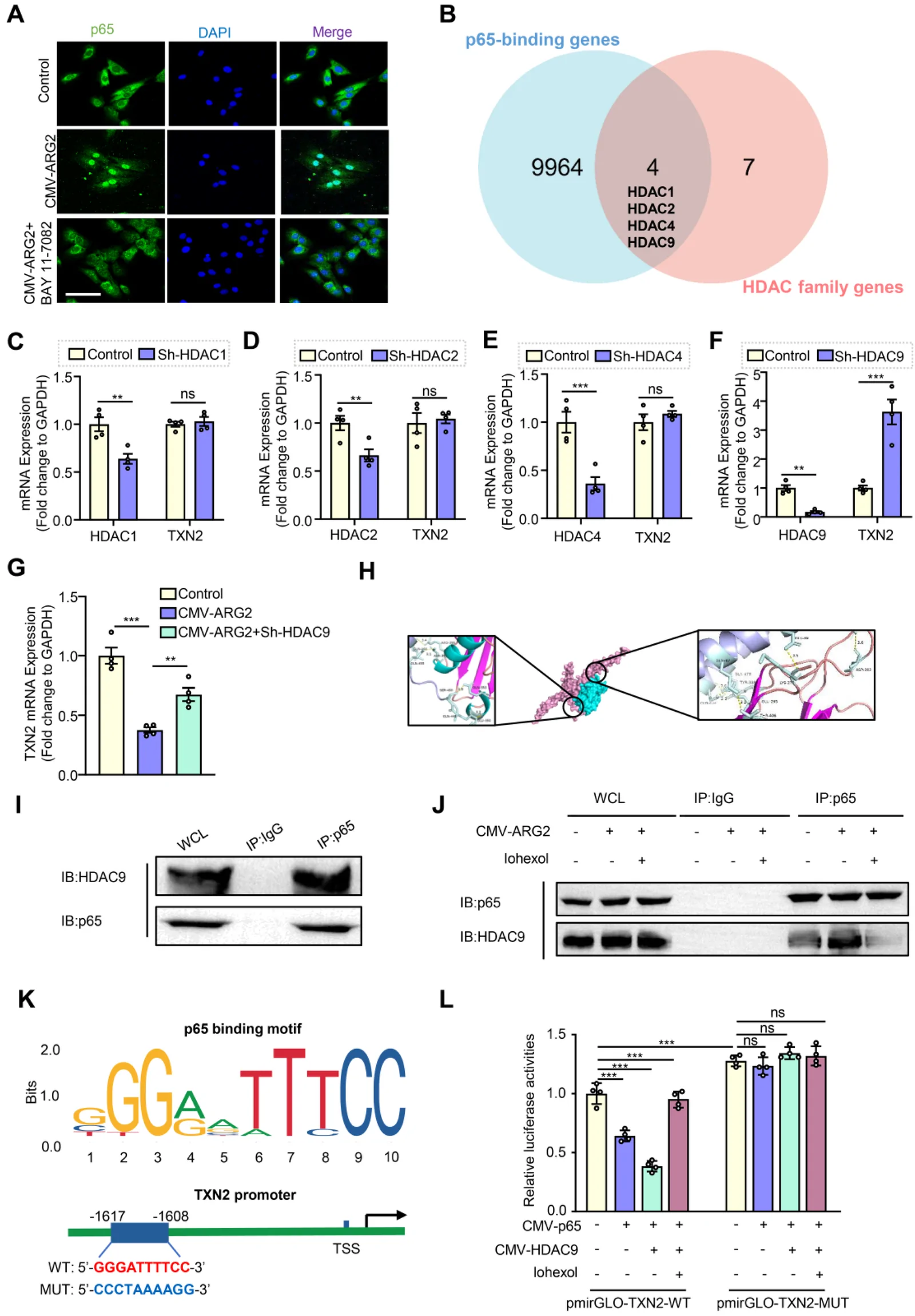
** Interaction of p65 with HDAC9 negatively regulates TXN2 expression.** MC3T3-E1 cells were transduced with pLKO.1-control (Con) or pLKO.1-HDAC1/2/4/9 shRNA lentiviral vectors. **(A)** Representative immunofluorescence images of p65 staining. Scale bar = 100 μm. **(B)** Venn diagram illustrating the overlap between p65-binding genes and HDAC family members. (C-F) qRT-PCR analysis of the mRNA expression levels of HDAC1 and TXN2 **(C)**, HDAC2 and TXN2 **(D)**, HDAC4 and TXN2 **(E)**, and HDAC9 and TXN2 **(F)**. **(G)** qRT-PCR analysis of TXN2 mRNA expression. **(H)** Molecular docking analysis predicting the interaction between p65 and HDAC9 proteins. **(I)** Co-immunoprecipitation followed by Western blot analysis of the interaction between p65 and HDAC9. MC3T3-E1 cells were transduced with pLJM1-empty vector (Con) or pLJM1-HA-ARG2 (CMV-ARG2) to induce ARG2 overexpression and subsequently treated with iohexol (25 mg/mL) for 24 h. **(J)** Co-immunoprecipitation and Western blot analysis of p65-HDAC9 interaction. **(K)** Schematic representation of the predicted human p65-binding motif within the TXN2 promoter and the corresponding luciferase reporter constructs. HEK-293T cells were co-transfected with wild-type (TXN2-WT) or p65-binding site-mutated (TXN2-MUT) TXN2 promoter luciferase reporter plasmids together with the indicated pLJM1-p65 and/or pLJM1-HDAC9 expression vectors for dual-luciferase reporter assays. **(L)** Quantitative analysis of dual-luciferase reporter activity. Statistical significance was determined using one-way ANOVA. Data are presented as mean ± SEM (n = 4). ns: not significant; *p < 0.05, **p < 0.01, ***p < 0.001 between the indicated groups.

**Figure 8:**
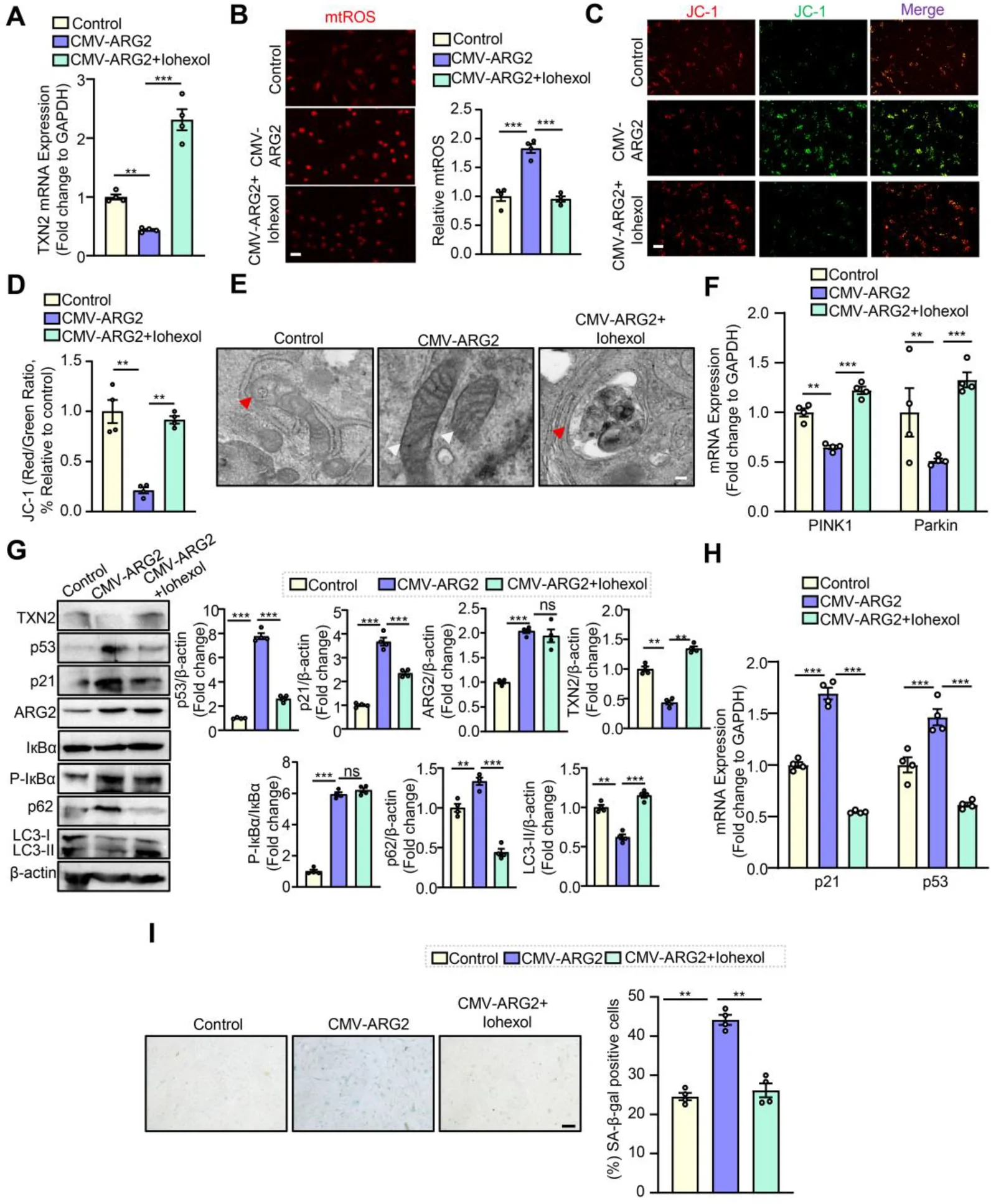
** Disruption of the p65-HDAC9 interaction alleviates ARG2-mediated mitochondrial dysfunction, mitophagy impairment and senescence in osteoblasts.** MC3T3-E1 cells were transduced with pLJM1-EGFP (Con) or pLJM1-HA-ARG2 (CMV-ARG2) to induce ARG2 overexpression, followed by treatment with iohexol (25 mg/mL) for 24 h. **(A)** qRT-PCR analysis of TXN2 mRNA expression. **(B)** Mitochondrial superoxide production was assessed by MitoSOX staining (1 μM). Scale bar = 100 μm. **(C)** Mitochondrial membrane potential (Δψm) was evaluated by JC-1 staining. Scale bar = 100 μm. **(D)** Quantitative JC-1 staining analysis. **(E)** Representative TEM images showing mitophagy. Red arrows indicate mitophagic vesicles, and white arrows indicate mitochondria. Scale bar = 500 nm. **(F)** qRT-PCR analysis of PINK1 and Parkin mRNA expression. **(G)** Immunoblotting analysis of TXN2, p21, p53, ARG2, p62, IκBα, phosphorylated IκBα (P-IκBα), and LC3 protein expression. Quantification of protein levels is shown in the bar graphs on the right. **(H)** qRT-PCR analysis of p21 and p53 mRNA expression. **(I)** SA-β-gal staining. Bar graphs represent the quantification of SA-β-gal-positive cells. Scale bar = 100 μm. Statistical significance was determined by one-way ANOVA. Data are presented as mean ± SEM (n = 4). ns, not significant; **p < 0.01, ***p < 0.001 between the indicated groups.

**Figure 9:**
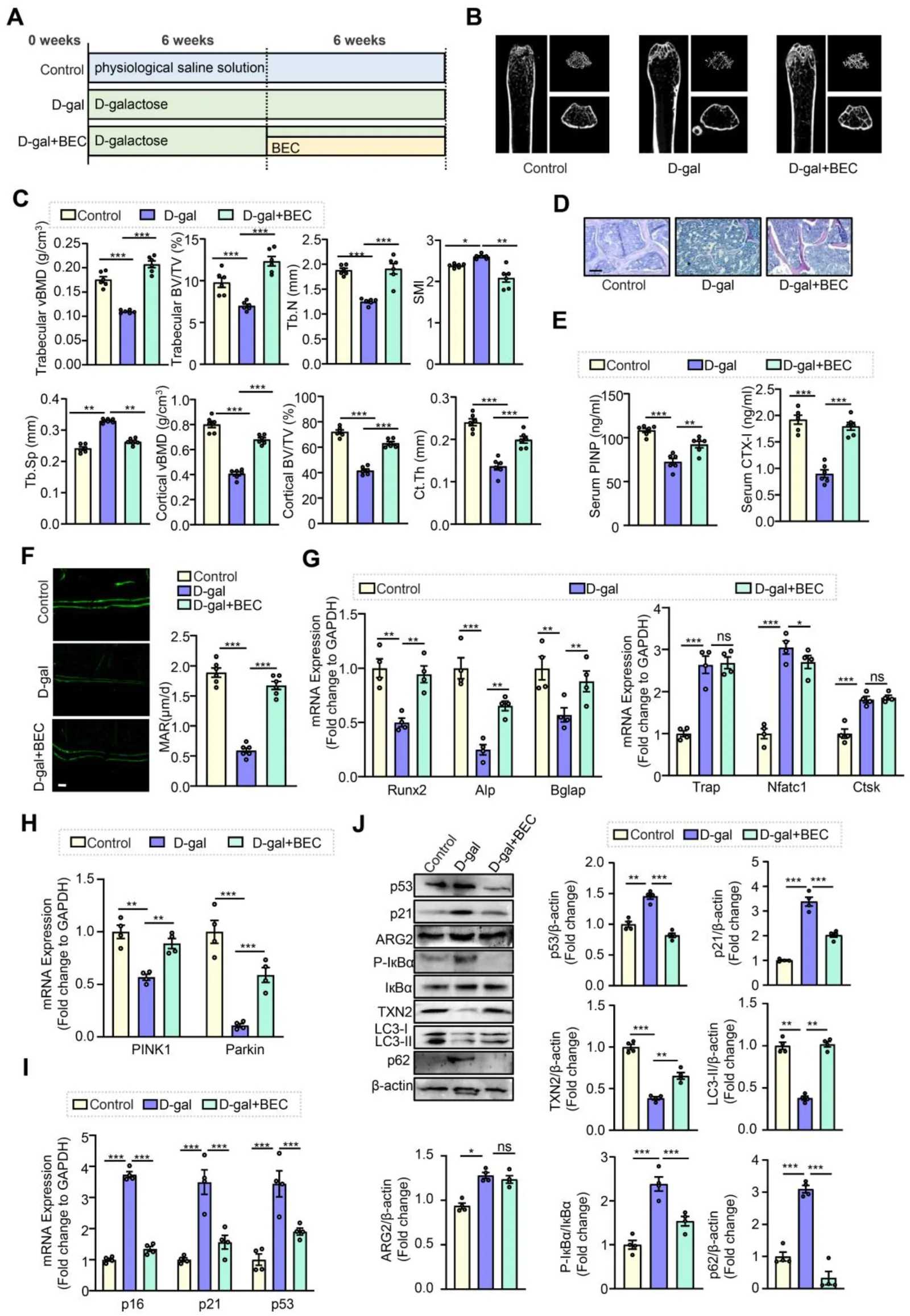
** BEC ameliorates D-galactose-induced osteoporosis in mice.** Mice in the D-gal group received D-galactose (1000 mg/kg/day) for 12 weeks. Mice in the D-gal + BEC group were treated with D-galactose (1000 mg/kg/day) for 6 weeks, followed by daily subcutaneous administration of BEC (2.3 mg/kg/day) for the remaining 6 weeks. Control mice received physiological saline for the same duration. **(A)** Schematic illustration of the experimental design and animal grouping. **(B)** Representative micro-CT images of vertebrae from 6-month-old control mice, D-galactose-treated mice, and D-galactose-treated mice receiving BEC (n = 6 per group). **(C)** Quantitative micro-CT analysis of trabecular and cortical volumetric bone mineral density (vBMD), trabecular bone volume/tissue volume (BV/TV), cortical BV/TV, structural model index (SMI), trabecular number (Tb.N), trabecular separation (Tb.Sp), and cortical thickness (Ct.Th) (n = 6 per group). **(D)** Representative H&E-stained images of decalcified vertebral sections from each group (n = 6 per group). Scale bar = 500 μm. **(E)** Serum levels of the bone turnover markers PINP and CTX-I, measured by ELISA (n = 6 per group). **(F)** Representative images of calcein double labeling in trabecular bone and quantitative analysis of the mineral apposition rate (MAR). Scale bar = 50 μm. **(G)** qRT-PCR analysis of mRNA expression levels of osteoblast- and osteoclast-related markers in vertebrae (n = 4 per group). **(H)** qRT-PCR analysis of PINK1 and Parkin mRNA expression in vertebrae (n = 4 per group). **(I)** qRT-PCR analysis of senescence-associated gene expression (n = 4 per group). **(J)** Immunoblot analysis of p21, p53, ARG2, IκBα, phosphorylated IκBα (p-IκBα), TXN2, p62, and LC3 protein expression. Quantitative densitometric analyses are shown in the corresponding bar graphs. Statistical significance was determined by one-way ANOVA. Data are presented as mean ± SEM. ns, not significant; *p < 0.05, **p < 0.01, ***p < 0.001 between the indicated groups.

## Abbreviations

AAOP: age-associated osteoporosis
ARG2: arginase 2
D-gal: D-galactose
ASBMR: American Society for Bone and Mineral Research
ObARG2-cKO: osteoblast-specific ARG2 knockout
OBs: osteoblasts
OCs: osteoclasts
mtROS: mitochondrial reactive oxygen species
ΔΨm: mitochondrial membrane potential
SD: standard deviation
TXN2: thioredoxin 2
ROS: reactive oxygen species
VMSCs: vascular smooth muscle cells
AOPPs: advanced oxidation protein products
BMD: bone mineral density
vBMD: volumetric bone mineral density
BV/TV: bone volume
Tb.N: trabecular number
Tb.Sp: trabecular separation
SMI: structure model index
PINP: procollagen I N-terminal propeptide
MAR: mineral apposition rate
CTX-I: C-terminal cross-linked telopeptide of type I collagen
HE: hematoxylin–eosin
BMSCs: bone marrow mesenchymal stem cells
BMMs: bone marrow mononuclear cells
M-CSF: macrophage colony-stimulating factor
*Oc-Cre*: *Osteocalcin-Cre* mice
Baf A1: bafilomycin A1
ARS: alizarin red S staining
ALP: alkaline phosphatase
Rapa: rapamycin
TEM: transmission electron microscopy
NAC: N-acetylcysteine
BEC: S-(2-boronoethyl)-L-cysteine
MOSRGs: mitochondrial oxidative stress-related genes
MRGs: mitophagy-related genes
OSRGs: osteoporosis-related genes
ARG2RGs: ARG2-related genes
SRGs: senescence-related genes
Cdkn1a: cyclin dependent kinase inhibitor 1a
FAS: fas cell surface death receptor
CAT: catalase
TXN2RGs: TXN2-related genes
HDAC: histone deacetylase
HDAC9: histone deacetylase 9
SASP: senescence-associated secretory phenotype
AGEs: advanced glycation end-products
ARG1: arginase
