# Supplementary material for: ARG2 drives osteoblast senescence by repressing mitophagy via the IκB–NF-κB–TXN2 signaling axis: implications for age-associated osteoporosis
Source: Theranostics. 2026 Sep 18;16(16):9295–321. doi: 10.7150/thno.129818 (PMC13684819; doi:10.7150/thno.129818)
Supplement: Supplementary file 1 [file thnov16p9295s1.pdf]

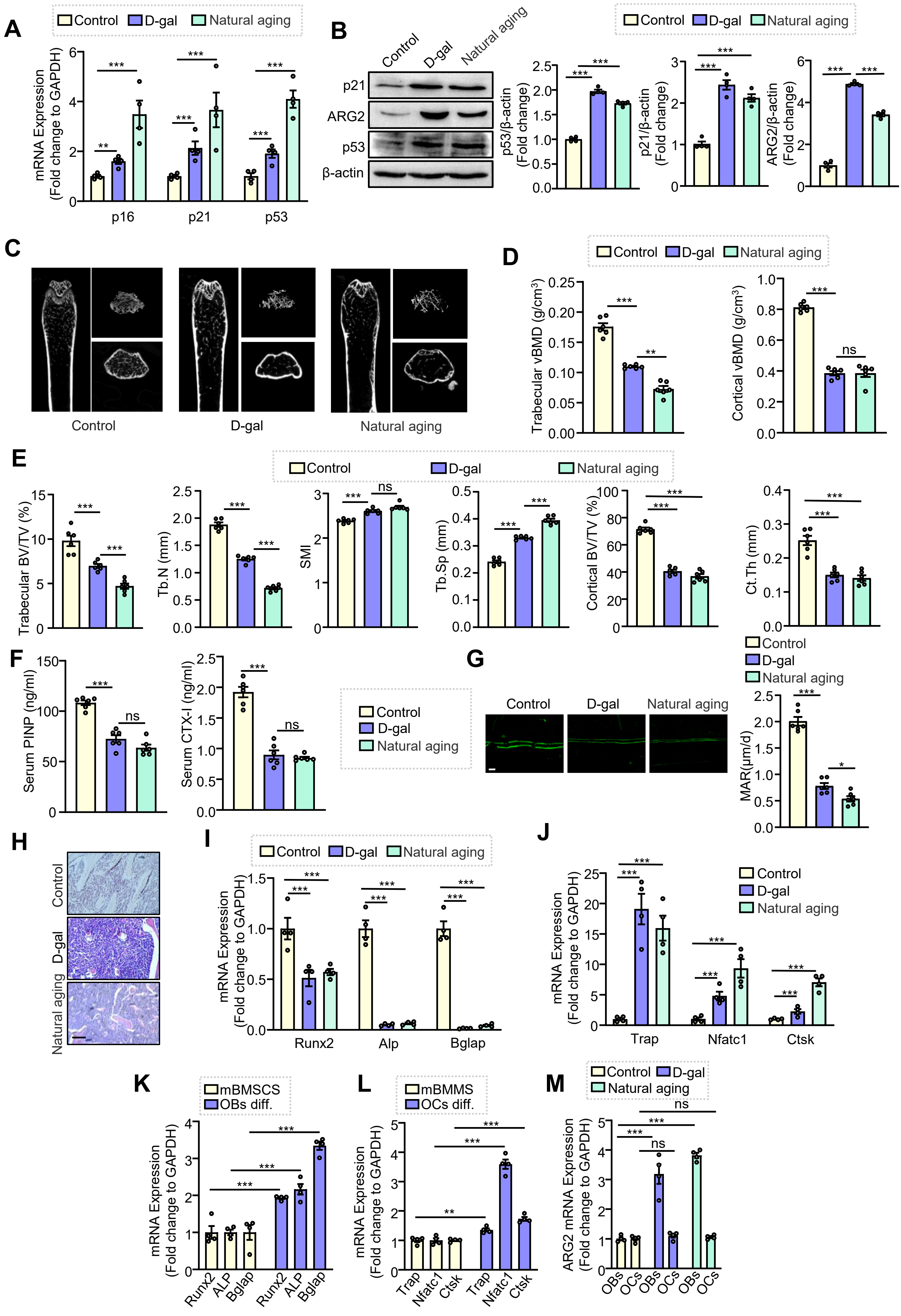
A
Control
D-gal
Natural aging
mRNA Expression
(Fold change to GAPDH)
p16
p21
p53
B
Control
D-gal
Natural aging
p21
ARG2
p53
β-actin
p53/β-actin
(Fold change)
p21/β-actin
(Fold change)
ARG2/β-actin
(Fold change)
C
Control
D-gal
Natural aging
D
Trabecular vBMD (g/cm³)
Cortical vBMD (g/cm³)
E
Trabecular BV/TV (%)
Tb.N (mm)
SMI
Tb.Sp (mm)
Cortical BV/TV (%)
Ct.Th (mm)
F
Serum PINP (ng/ml)
Serum CTX-I (ng/ml)
G
MAR(μm/d)
H
I
Runx2
Alp
Bglap
J
Trap
Nfatc1
Ctsk
K
mBMSCS
OBs diff.
Runx2
ALP
Bglap
L
mBMMS
OCs diff.
Trap
Nfatc1
Ctsk
M
ARG2 mRNA Expression
(Fold change to GAPDH)
OBs
OCs

Supplementary Figure S1. Osteoporotic phenotypes in D-galactose-induced and naturally aged mouse models. Mice in the D-gal group received D-galactose (1000 mg/kg/day) for 12 weeks. (A) qRT-PCR analysis of senescence-associated gene expression in vertebral tissues (n = 4 per group). (B) Immunoblot analysis of p21, p53 and ARG2 protein expression in vertebrae; corresponding densitometric quantification is shown on the right (n = 4 per group). (C) Representative micro-CT images of vertebrae from 6-month-old control mice, D-galactose-treated mice, and 20-month-old naturally aged mice (n = 6 per group). (D) Quantitative micro-CT assessment of trabecular and cortical volumetric bone mineral density (vBMD) (n = 6 per group). (E) Quantitative analysis of trabecular bone volume fraction (BV/TV), cortical BV/TV, structural model index (SMI), trabecular number (Tb.N), trabecular separation (Tb.Sp), and cortical thickness (Ct.Th) determined by micro-CT (n = 6 per group). (F) Serum levels of the bone turnover markers PINP and CTX-I measured by ELISA (n = 6 per group). (G) Representative images of calcein double labeling in trabecular bone and quantification of mineral apposition rate (MAR). Scale bar = 50 μm. (H) Representative hematoxylin and eosin (H&E)-stained decalcified vertebral sections from 6-month-old control mice, D-galactose-treated mice, and 20-month-old naturally aged mice (n = 6 per group). Scale bar = 500 μm. (I, J) qRT-PCR analysis of osteoblast-related (I) and osteoclast-related (J) gene expression in vertebrae (n = 4 per group). (K, L) qRT-PCR analysis of osteoblast-related (K) and osteoclast-related (L) gene expression in vertebrae (n = 4 per group). (M) qRT-PCR analysis of ARG2 mRNA expression in vertebrae (n = 4 per group). Statistical significance was determined by one-way ANOVA. Data are presented as mean ± SEM. ns, not significant; $^{**}p < 0.01$, $^{***}p < 0.001$ versus the indicated groups.

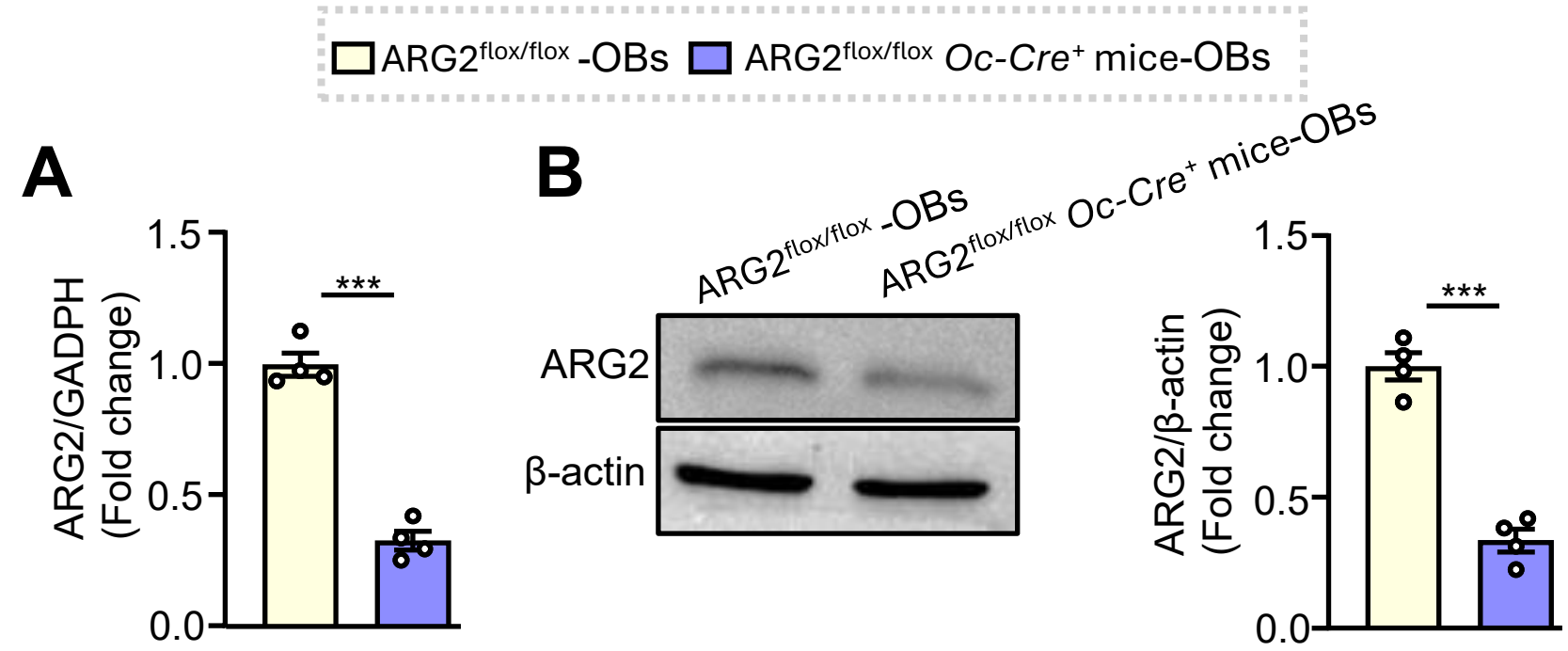

Supplementary Figure S2. Validation of osteoblast-specific ARG2 deletion in primary osteoblasts. Primary osteoblasts isolated from ARG2$^{flox/flox}$ mice and osteoblast-specific ARG2 knockout (ARG2$^{flox/flox}$ *Oc-Cre*$^{+}$) mice were subjected to Western blot and qRT-PCR analyses. **A.** Relative ARG2 mRNA levels in primary osteoblasts. **B.** Representative Western blot and quantitative analysis of ARG2 protein levels in primary osteoblasts. Statistical significance was determined by one-way ANOVA. Data are presented as mean ± SEM. n = 4. ***$p < 0.001$ between the indicated groups.

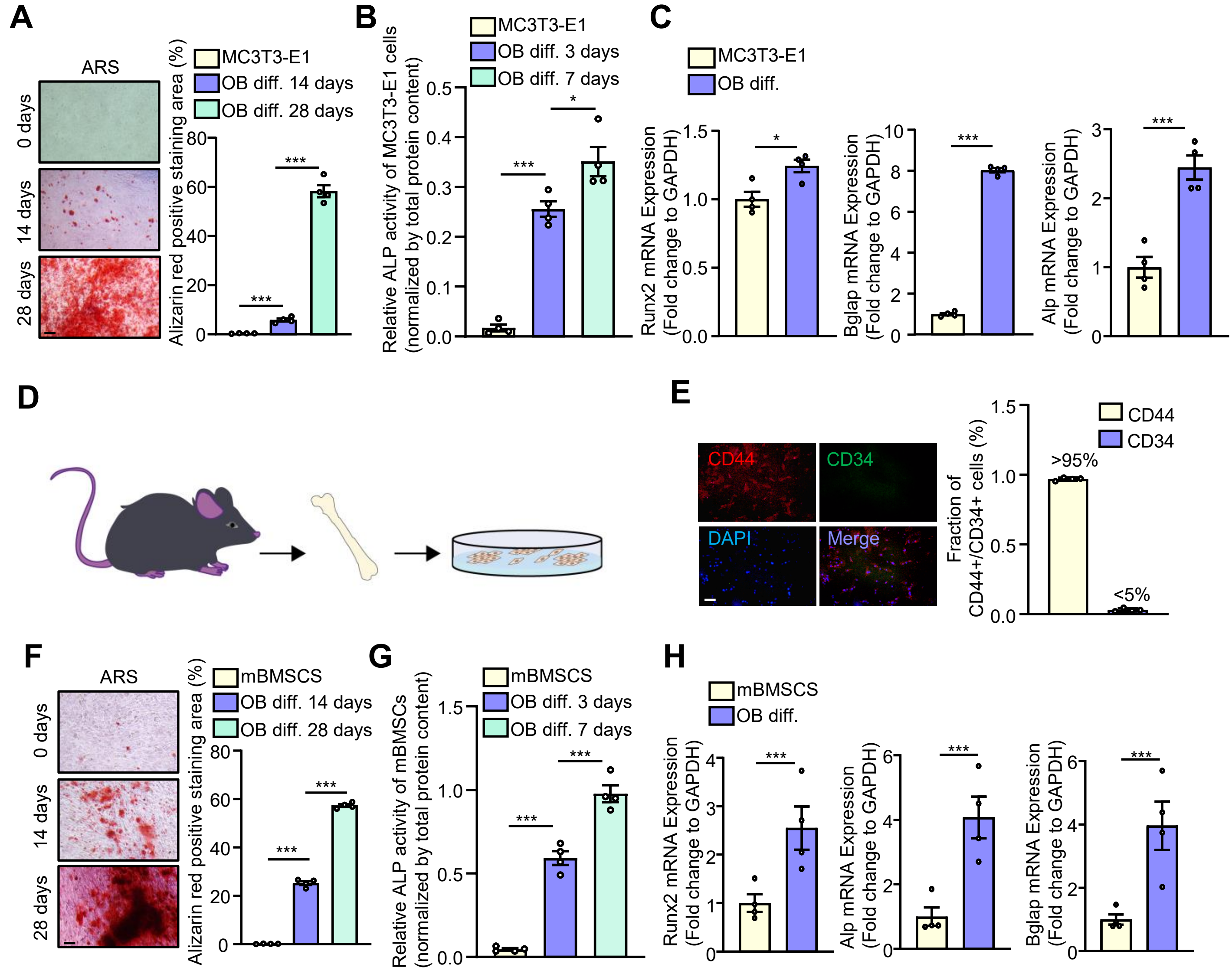

Supplementary Figure S3. Osteogenic differentiation of mesenchymal progenitor cells into osteoblasts. (A) Representative images of Alizarin red S staining (ARS) and corresponding quantification of mineralized nodules. (B) Alkaline phosphatase (ALP) activity assay. (C) qRT-PCR analysis of the mRNA expression levels of Runx2, Bglap, and Alp. (D) Schematic illustration of the isolation and extraction of bone marrow-derived mesenchymal stem cells (BMSCs) from murine femurs. (E) Representative immunofluorescence images of CD44 and CD34 staining. Quantification of positively stained cells is shown on the right. (F) Representative images of ARS and corresponding quantification of mineralized nodules. (G) ALP activity assay. (H) qRT-PCR analysis of the mRNA expression levels of Runx2, Bglap, and Alp. Statistical significance was determined by one-way ANOVA. Data are presented as mean ± SEM. n = 4. *$p < 0.05$, ***$p < 0.001$ between the indicated groups. Scale bar = 100 μm.

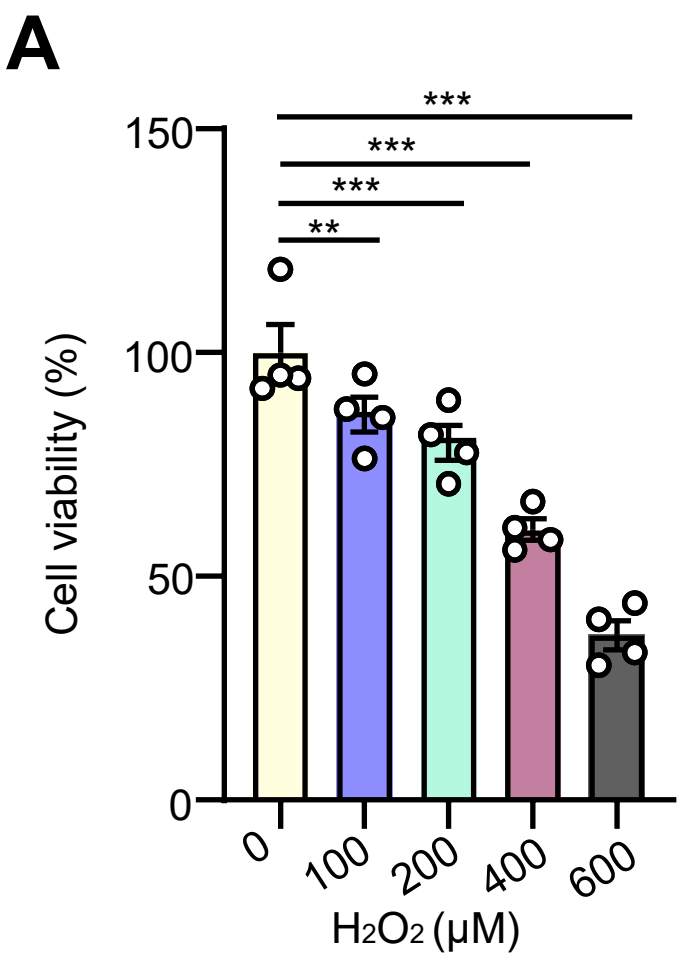

Supplementary Figure S4. Determination of the optimal H_2O_2 concentration for establishing the osteoblast senescence model. (A) MC3T3-E1 cells were treated with increasing concentrations of H_2O_2 (0, 100, 200, 400, and 600 μM) for 24 h, followed by assessment of cell viability using the CCK-8 assay. Data are presented as the mean ± SD (n = 4). **P < 0.01, ***P < 0.001 versus the control group.

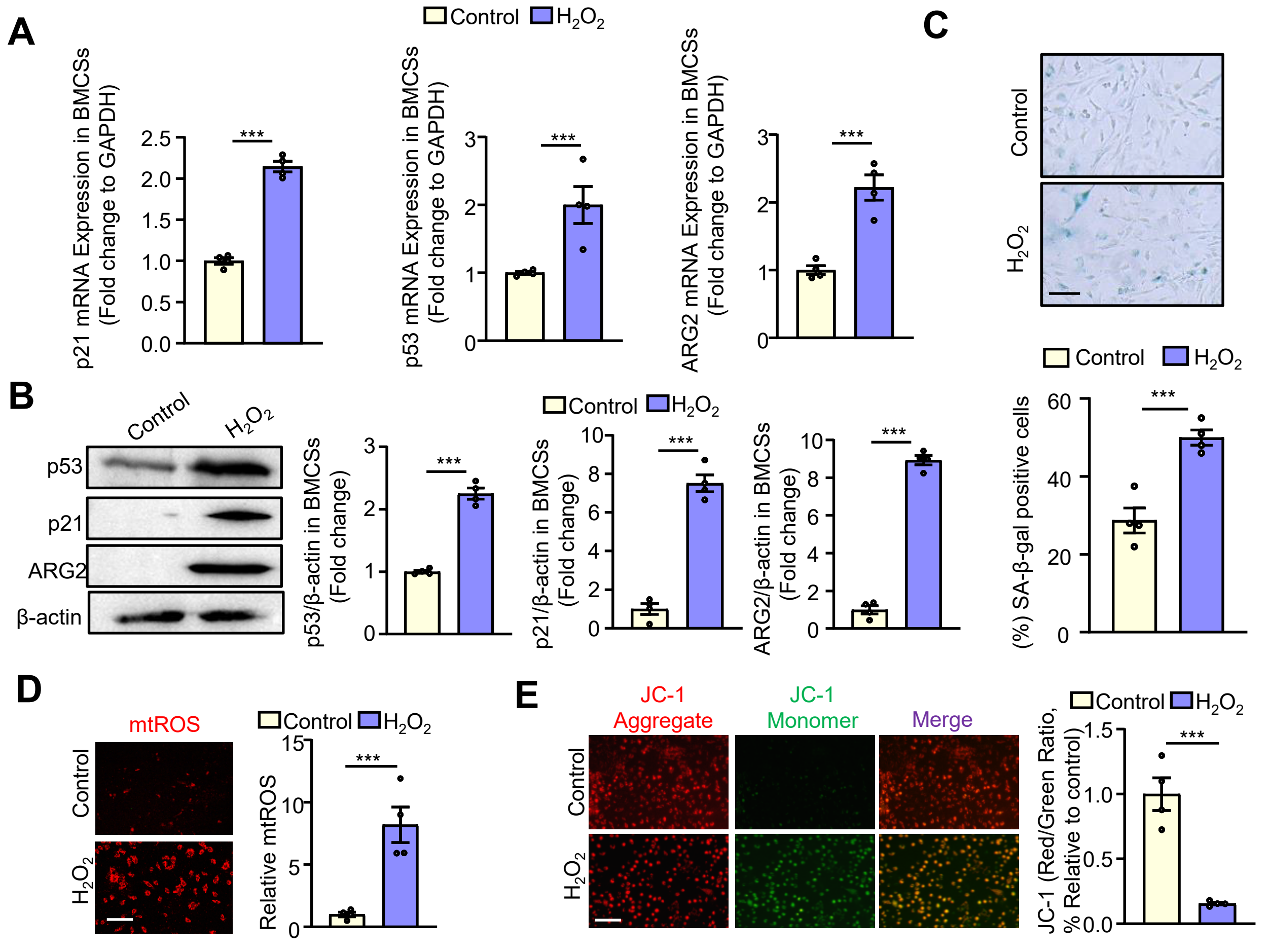

Supplementary Figure S5. **H_2O_2 promotes mitochondrial dysfunction and senescence in primary BMSC-derived osteoblasts.** Primary BMSC-derived osteoblasts were treated with H_2O_2 (200 μM) for 24 h. (A) qRT-PCR analysis of p21, p53, and ARG2 mRNA expression. (B) Immunoblot analysis of p21, p53, and ARG2 protein levels, with corresponding densitometric quantification shown in the right panels. (C) Senescence-associated β-galactosidase (SA-β-gal) staining and quantification of SA-β-gal-positive cells. (D) Mitochondrial superoxide production assessed by MitoSOX staining (1 μM), with quantitative analysis shown in the right panels. (E) Mitochondrial membrane potential (Δψm) evaluated by JC-1 staining, with quantitative analysis shown in the right panels. Statistical significance was determined by one-way ANOVA. Data are presented as mean ± SEM (n = 4). ***$p < 0.001$ between the indicated groups. Scale bar = 100 μm.

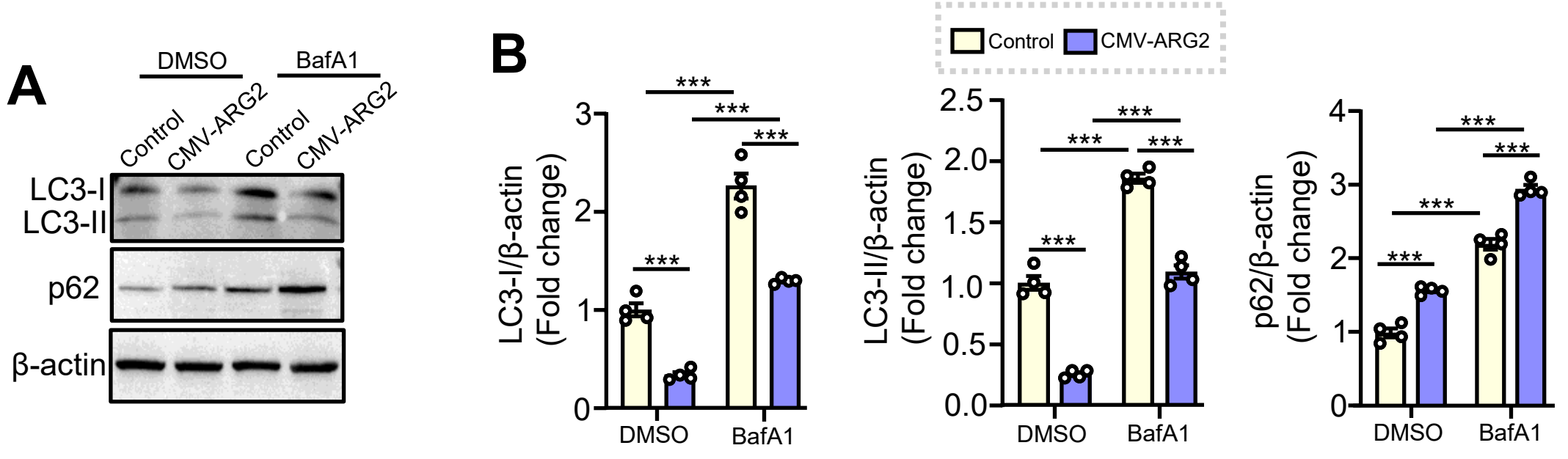

Supplementary Figure S6. ARG2 overexpression impairs PINK1/Parkin-mediated mitophagy and alters mitophagic flux in osteoblasts. MC3T3-E1 cells were transduced with pLJM1-EGFP (Con) or pLJM1-HA-ARG2 (CMV-ARG2) and subsequently treated with bafilomycin A1 (Baf A1, 100 nM) for 4 h.(A) Representative Western blot images showing LC3-I, LC3-II, and p62 expression under the indicated conditions. (B) Quantification of LC3 and p62 protein levels. Statistical significance was determined by one-way ANOVA. Data are presented as mean ± SEM (n = 4). ns: not significant, ***p < 0.001 between the indicated groups.

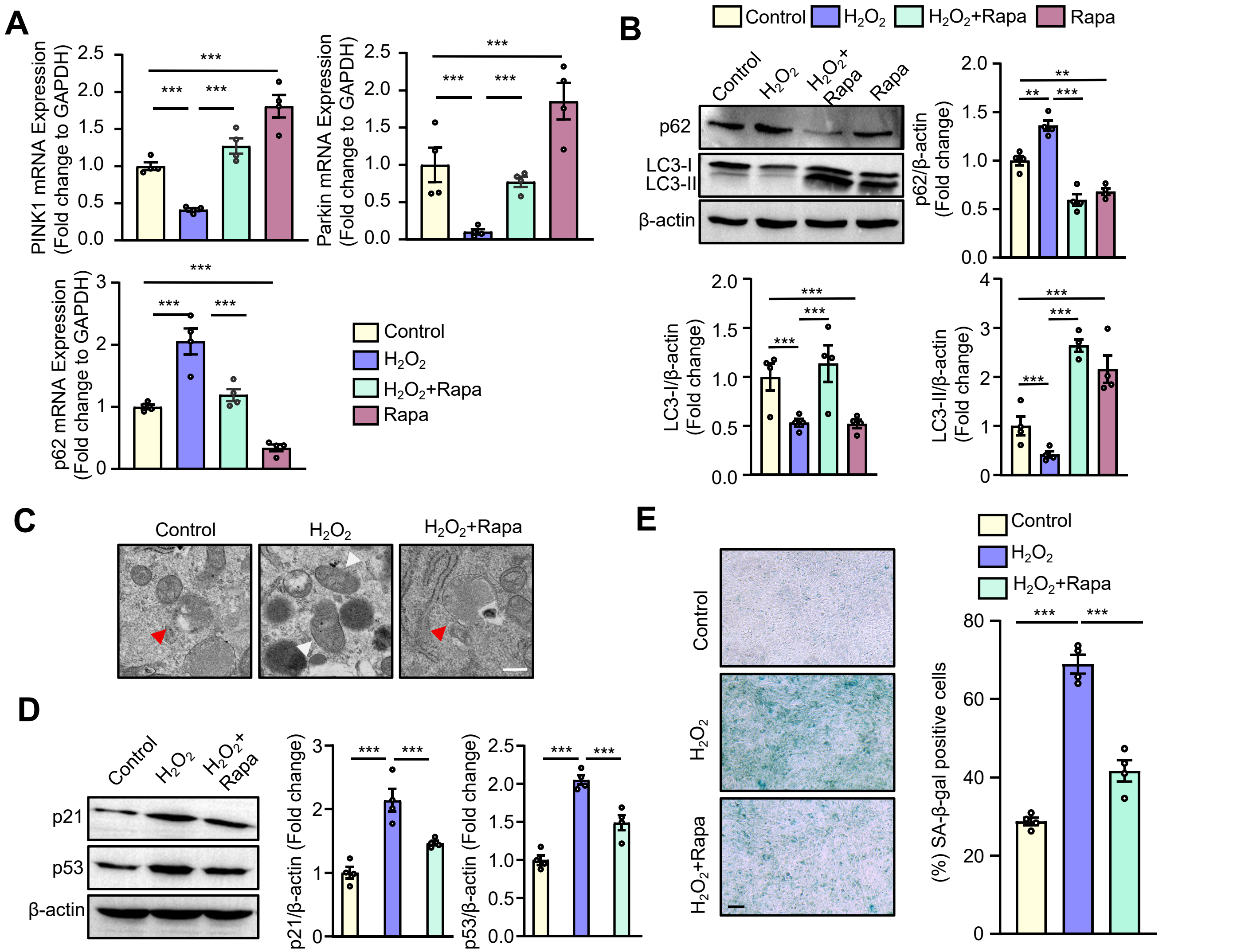

Supplementary Figure S7. Restoration of mitophagy by rapamycin attenuates H_2O_2-induced cellular senescence. MC3T3-E1 cells were treated with H_2O_2 (200 μM) for 24 h, followed by rapamycin (Rapa, 100 nM) treatment for an additional 24 h. (A) qRT-PCR analysis of the mRNA expression levels of PINK1, Parkin, and p62. (B) Immunoblotting analysis of p62 and LC3 protein expression, with corresponding densitometric quantification shown in the bar graphs on the right. (C) Representative TEM images showing mitophagy. Red arrows indicate mitophagic vesicles, and white arrows indicate mitochondria. Scale bar = 500 nm. (D) Immunoblotting analysis of p21 and p53 protein expression, with corresponding densitometric quantification shown in the bar graphs on the right. (E) Senescence-associated β-galactosidase (SA-β-gal) staining. Bar graphs show the quantification of SA-β-gal-positive cells. Statistical significance was determined by one-way ANOVA. Data are presented as mean ± SEM (n = 4). $^{**}p < 0.01$, $^{***}p < 0.001$ between the indicated groups.

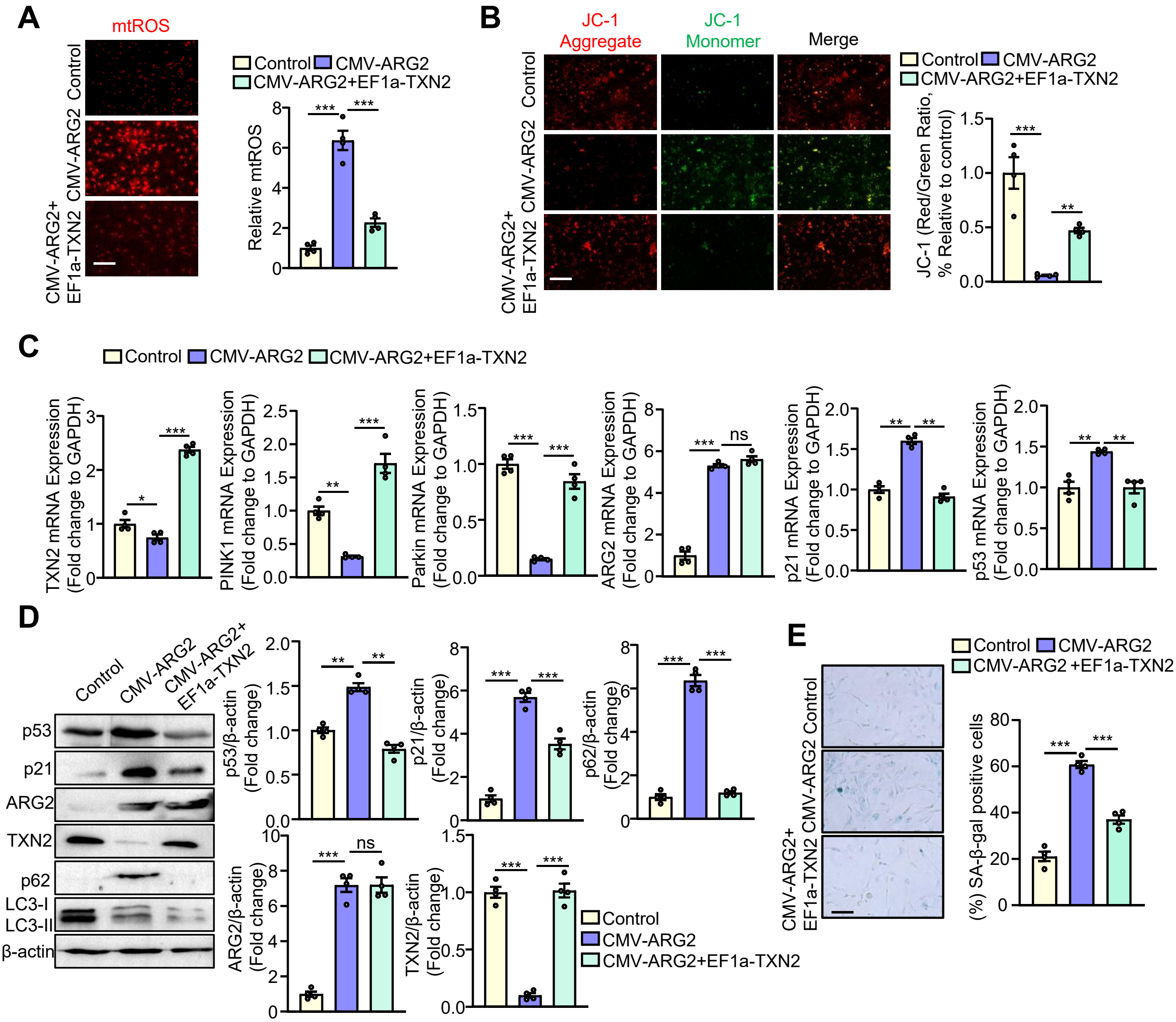

Supplementary Figure S8. Overexpression of TXN2 alleviated ARG2-induced mitochondrial oxidative stress, mitophagy impairment, and cellular senescence in primary BMSC-derived osteoblasts. Primary BMSC-derived osteoblasts were transduced with pLJM1-EGFP (Con) or pLJM1-HA-ARG2 (CMV-ARG2) to induce ARG2 overexpression, followed by transduction with pLJM1-HA-TXN2 (EF1a-TXN2) to overexpress TXN2. (A) Mitochondrial superoxide production was assessed by staining with 1 μM MitoSOX, with quantitative analysis shown in the right panels. (B) Mitochondrial membrane potential (Δψm) was evaluated using JC-1 staining, with quantitative analysis shown in the right panels. (C) qRT-PCR analysis of the mRNA expression levels of p21, p53, ARG2, TXN2, PINK1, and Parkin. (D) Immunoblot analysis of p21, p53, ARG2, TXN2, p62, and LC3 protein expression. Quantification of protein levels is shown in the bar graphs on the right. (E) Senescence-associated β-galactosidase (SA-β-gal) staining. Bar graphs represent the quantification of SA-β-gal-positive cells. Statistical significance was determined by one-way ANOVA. Data are presented as mean ± SEM (n = 4); *p < 0.05, **p < 0.01, ***p < 0.001 between the indicated groups. Scale bar = 100 μm.

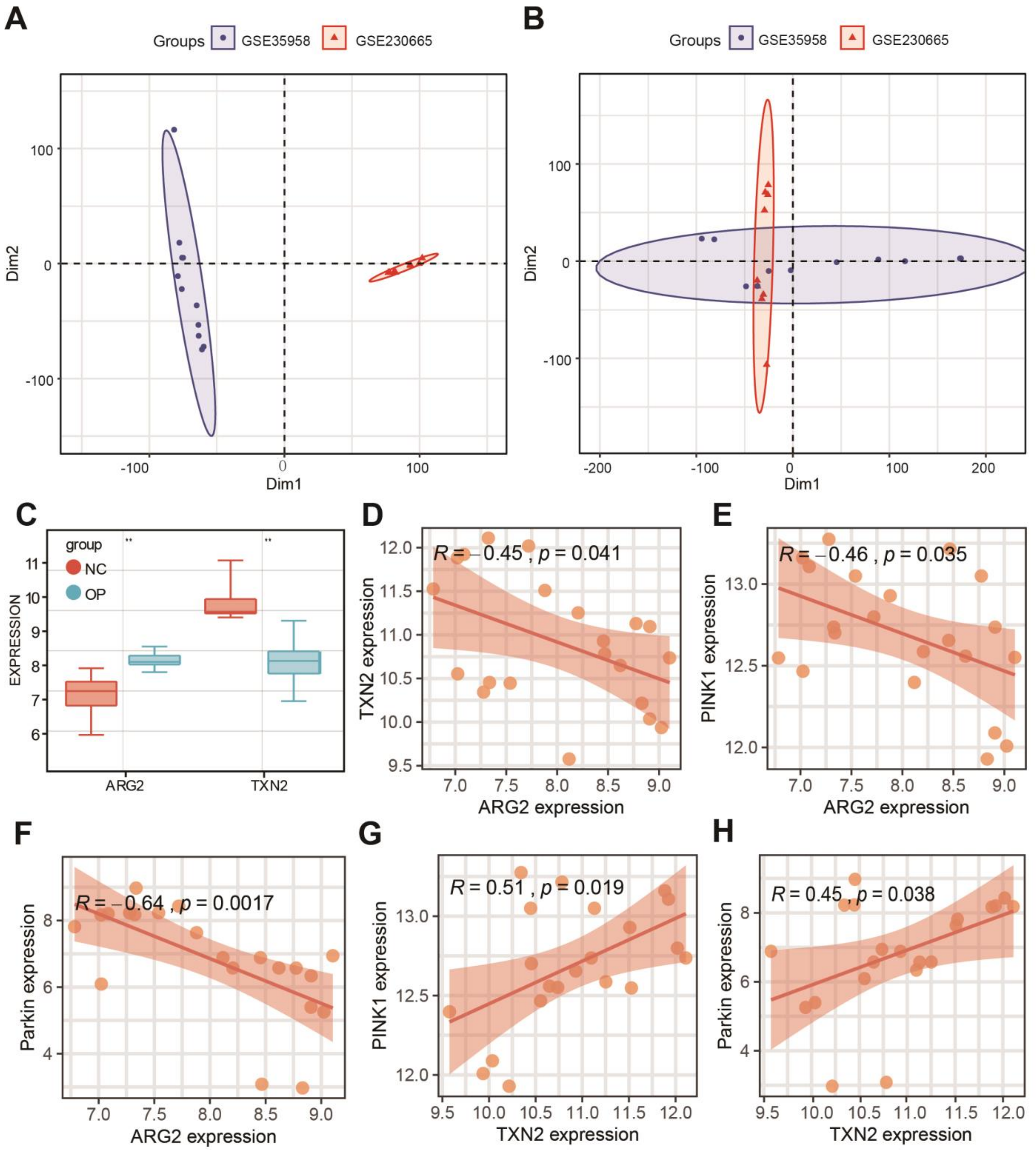

Supplemental Figure S9. Expression profiles of ARG2 and TXN2 and their Pearson correlation analyses with PINK1 and Parkin in osteoporotic patients. (A-B) Principal component analysis (PCA) plots of human osteoporosis samples from the GEO datasets GSE35958 and GSE230665 before (A) and after (B) batch effect correction, demonstrating effective elimination of inter-dataset technical variation while preserving biological heterogeneity. (C) Differential expression analysis of ARG2 and TXN2 between non-osteoporotic normal control (NC) and osteoporotic (OP) groups in the batch-corrected integrated dataset. Data are presented as median with interquartile range (IQR), and statistical significance was assessed using a two-sided Wilcoxon rank-sum test (**$p < 0.01$). (D-F) Pearson correlation analyses between ARG2 expression and TXN2 (D), PINK1 (E) or Parkin (F). (G-H) Pearson correlation analyses between TXN2 expression and PINK1 (G) or Parkin (H). Each dot represents an individual sample. Solid lines indicate linear regression fits, and shaded areas represent 95% confidence intervals. Pearson correlation coefficients (r) and corresponding p values are shown in each panel.

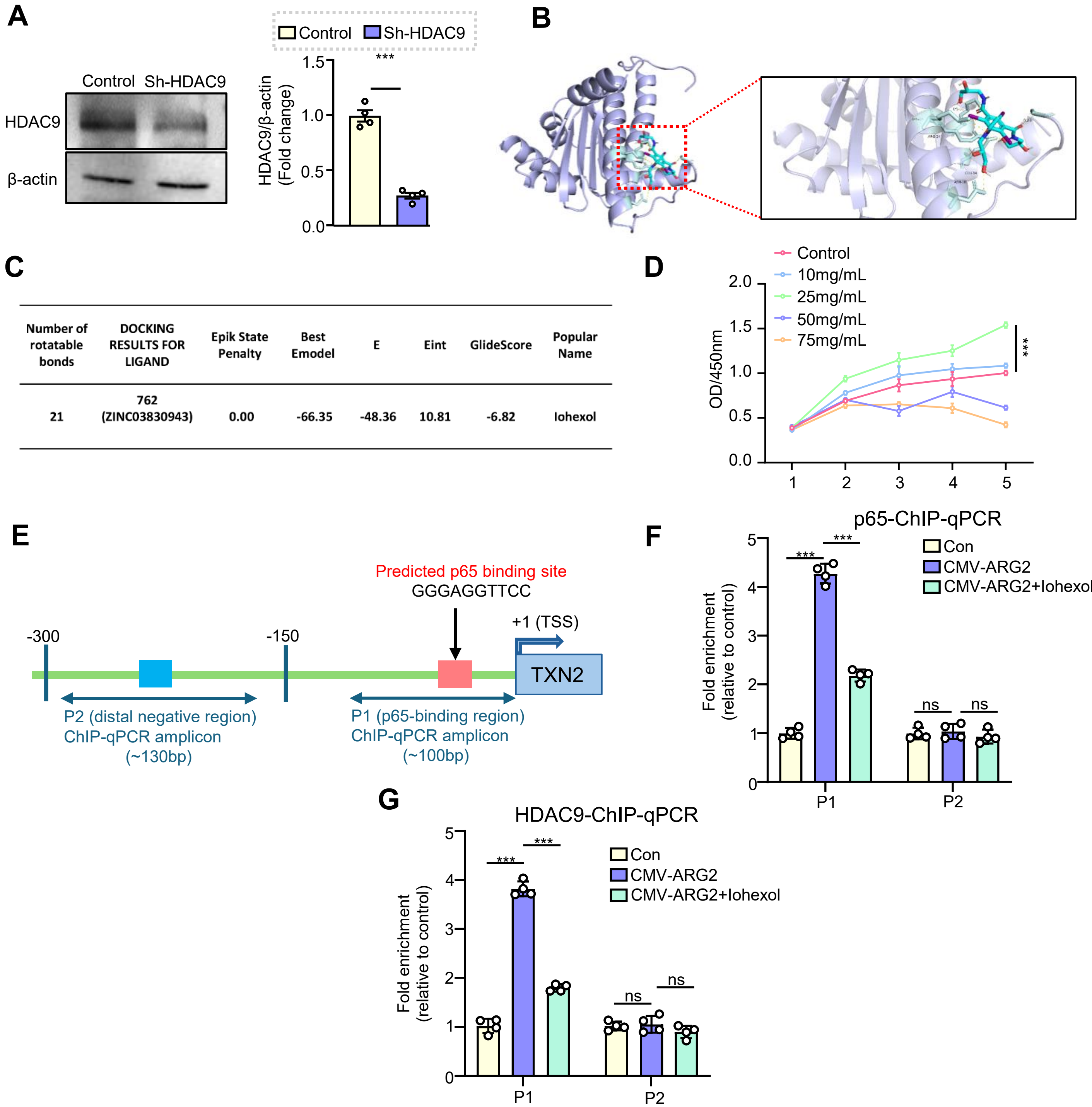

Number of rotatable bonds	DOCKING RESULTS FOR LIGAND	Epik State Penalty	Best Emodel	E	Eint	GlideScore	Popular Name
21	762 (ZINC03830943)	0.00	-66.35	-48.36	10.81	-6.82	Iohexol

Supplementary Figure S10. Identification of HDAC9 as a critical regulator of TXN2 transcription and screening of iohexol as a potential inhibitor of the p65–HDAC9 interaction. MC3T3-E1 cells were transduced with pLKO.1-control (Con) or pLKO.1-HDAC9 shRNA lentiviral vectors. (A) Representative Western blot images and quantitative analysis confirming the efficiency of HDAC9 knockdown (B) Molecular docking analysis illustrating the competitive binding of Iohexol (0-75 mg/mL) and p65 to HDAC9. (C) In silico screening and prediction of candidate compounds targeting the p65-HDAC9 binding interface. (D) MC3T3-E1 cells were treated with increasing concentrations of Iohexol (0, 10, 25, 50, and 75 mg/mL) for 24 h, followed by assessment of cell viability using the CCK-8 assay. MC3T3-E1 cells were transduced with pLJM1- EGFP (Con) or pLJM1-HA-ARG2 (CMV-ARG2) to induce ARG2 overexpression and subsequently treated with iohexol (25 mg/mL) for 24 h. (E) Schematic representation of the mouse TXN2 promoter showing the predicted NF-κB/p65-binding motif and the region amplified by ChIP-qPCR primers. (F) ChIP-qPCR analysis of p65 enrichment at the predicted p65-binding region (P1) and distal negative region (P2) of the TXN2 promoter. (G) ChIP-qPCR analysis of HDAC9 enrichment at the same TXN2 promoter region. Statistical significance was determined by one-way ANOVA. Data are presented as mean ± SEM (n = 4). ***$p < 0.001$ between the indicated groups.

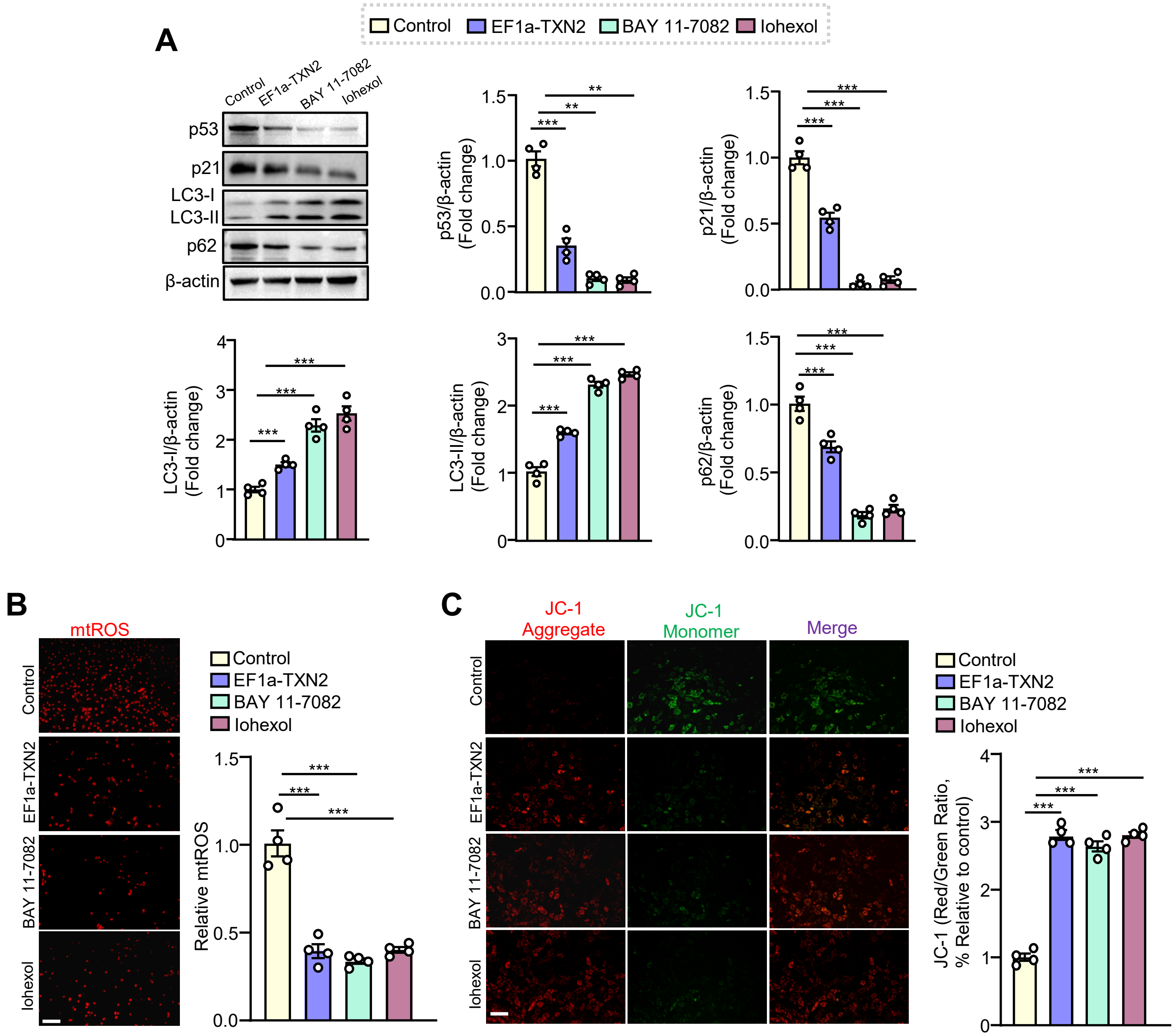

Supplementary Figure S11. TXN2 overexpression attenuates mitochondrial oxidative stress, mitochondrial dysfunction, osteoblast senescence, and autophagy dysregulation in primary osteoblasts from naturally aged mice. Primary osteoblasts were isolated from naturally aged mice and transduced with pLJM1-EGFP as control (Con) or pLJM1-HA-TXN2 (EF1α-TXN2) to overexpress TXN2, followed by treatment with BAY 11-7082 (10 nM) or iohexol (25 mg/mL) for 24 h. (A) Western blot analysis of p21, p53, LC3-I/LC3-II, and p62 protein expression to assess osteoblast senescence and autophagy. Quantification of protein levels is shown in the corresponding bar graphs. (B) Mitochondrial superoxide production was assessed by MitoSOX staining (1 μM). Scale bar = 100 μm. (C) Mitochondrial membrane potential (Δψm) was evaluated by JC-1 staining. Scale bar = 100 μm. Statistical significance was determined by one-way ANOVA. Data are presented as mean ± SEM (n = 4). ns, not significant; $^{**}p < 0.01$, $^{***}p < 0.001$ between the indicated groups.

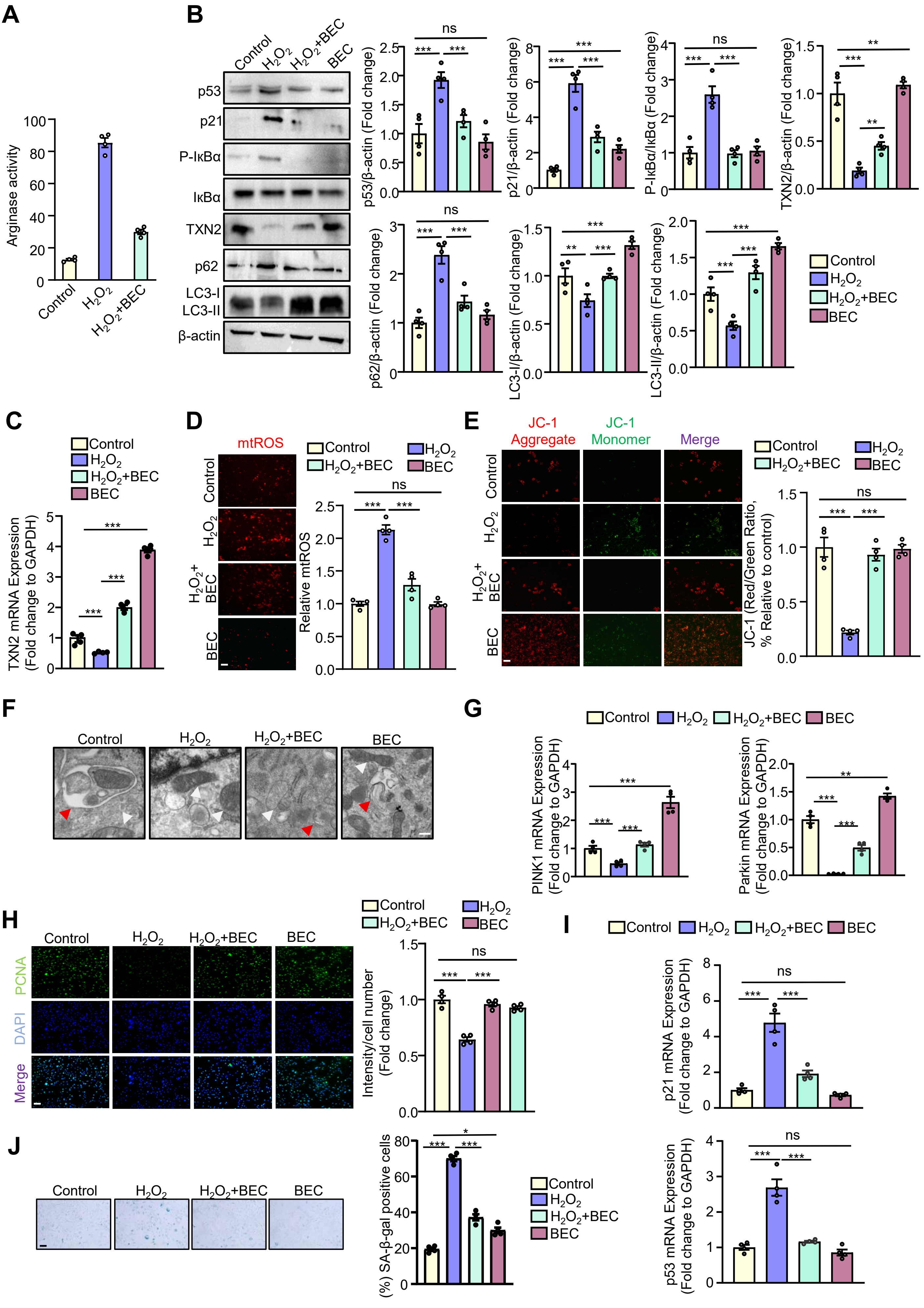

A
Arginase activity
Control
H2O2
H2O2+BEC
B
Control
H2O2
H2O2+BEC
BEC
p53
p21
P-IκBα
IκBα
TXN2
p62
LC3-I
LC3-II
β-actin
p53/β-actin (Fold change)
p21/β-actin (Fold change)
P-IκBα/IκBα (Fold change)
TXN2/β-actin (Fold change)
p62/β-actin (Fold change)
LC3-I/β-actin (Fold change)
LC3-II/β-actin (Fold change)
ns
C
TXN2 mRNA Expression (Fold change to GAPDH)
D
mtROS
Relative mtROS
E
JC-1 Aggregate
JC-1 Monomer
Merge
JC-1 (Red/Green Ratio, % Relative to control)
F
G
PINK1 mRNA Expression (Fold change to GAPDH)
Parkin mRNA Expression (Fold change to GAPDH)
H
PCNA
DAPI
Merge
Intensity/cell number (Fold change)
I
p21 mRNA Expression (Fold change to GAPDH)
p53 mRNA Expression (Fold change to GAPDH)
J
(%) SA-β-gal positive cells

Supplementary Figure S12. BEC ameliorates H_2O_2-induced mitochondrial oxidative stress, mitophagy impairment, and cellular senescence in osteoblasts. MC3T3-E1 cells were exposed to H_2O_2 (200 μM) for 24 h, followed by treatment with BEC (200 μM) for an additional 24 h. (A) Arginase activity assay. (B)Immunoblot analysis of p21, p53, IκBα, phosphorylated IκBα (P-IκBα), TXN2, p62, and LC3 protein expression, with corresponding densitometric quantification shown on the right. (C) qRT-PCR analysis of TXN2 mRNA expression. (D) Mitochondrial superoxide production assessed by MitoSOX staining (1 μM),with quantitative analysis shown in the right panels. Scale bar = 100 μm. (E) Mitochondrial membrane potential (Δψm) evaluated by JC-1 staining, with quantitative analysis shown in the right panels. Scale bar =100 μm. (F) Representative transmission electron microscopy (TEM) images showing mitophagy. Red arrows indicate mitophagic vesicles, and white arrows indicate mitochondria. Scale bar = 500 nm. (G) qRTPCR analysis of PINK1 and Parkin mRNA expression. (H) Representative images of PCNA immunofluorescence staining, with quantification of PCNA-positive cells shown on the right. Scale bar = 100 μm. (I) qRT-PCR analysis of p21 and p53 mRNA expression. (J) Senescence-associated β-galactosidase (SA-β-gal) staining, with quantification of SA-β-gal-positive cells shown in the accompanying bar graphs. Scale bar = 100 μm. Data are presented as mean ± SEM (n = 4). Statistical significance was determined by one-way ANOVA. ns, not significant; $^{**}p < 0.01$, $^{***}p < 0.001$ between the indicated groups.

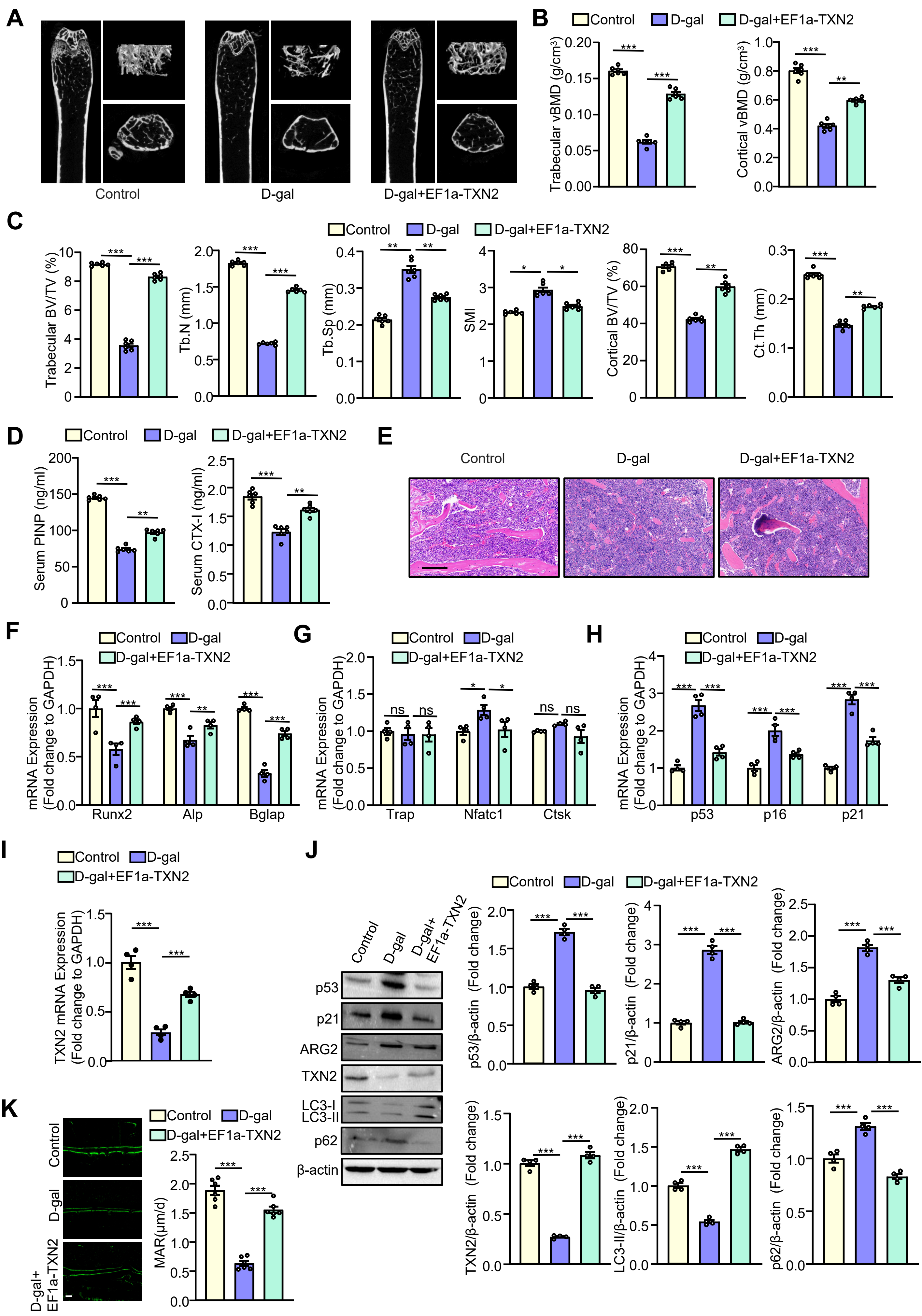

A
Control
D-gal
D-gal+EF1a-TXN2
B
Trabecular vBMD (g/cm³)
Cortical vBMD (g/cm³)
C
Trabecular BV/TV (%)
Tb.N (mm)
Tb.Sp (mm)
SMI
Cortical BV/TV (%)
Ct.Th (mm)
D
Serum PINP (ng/ml)
Serum CTX-I (ng/ml)
E
F
mRNA Expression (Fold change to GAPDH)
Runx2
Alp
Bglap
G
Trap
Nfatc1
Ctsk
H
p53
p16
p21
I
TXN2 mRNA Expression (Fold change to GAPDH)
J
p53
p21
ARG2
TXN2
LC3-I
LC3-II
p62
β-actin
p53/β-actin (Fold change)
p21/β-actin (Fold change)
ARG2/β-actin (Fold change)
TXN2/β-actin (Fold change)
LC3-II/β-actin (Fold change)
p62/β-actin (Fold change)
K
MAR(μm/d)

Supplementary Figure S13. Overexpression of TXN2 ameliorates D-galactose-induced osteoporosis in mice. Mice in the D-gal group received D-galactose (1000 mg/kg/day) for 18 weeks. Mice in the D-gal + EF1α-TXN2 group were treated with D-galactose (1000 mg/kg/day) for 18 weeks and additionally received weekly tail-vein injections of TXN2-overexpressing lentivirus (EF1α-TXN2; 200 μL per injection) beginning at week 8. Control mice received physiological saline for the same duration. (A) Representative micro-computed tomography (micro-CT) images of vertebrae from 6-month-old control mice, D-galactose-treated mice, and D-galactose-treated mice receiving EF1α-TXN2 ($n = 6$ per group). (B) Quantitative analysis of trabecular and cortical volumetric bone mineral density (vBMD). (C) Quantitative micro-CT analysis of trabecular bone volume fraction (BV/TV), cortical BV/TV, structure model index (SMI), trabecular number (Tb.N), trabecular separation (Tb.Sp), and cortical thickness (Ct.Th) ($n = 6$ per group). (D) Serum levels of the bone turnover markers PINP and CTX-I, measured by ELISA ($n = 6$ per group). (E) Representative H&E-stained images of decalcified vertebral sections from the indicated groups ($n = 6$ per group). Scale bar = 500 μm. (F) qRT-PCR analysis of osteoblast-related gene expression in vertebrae ($n = 4$ per group). (G) qRT-PCR analysis of osteoclast-related gene expression in vertebrae ($n = 4$ per group). (H) qRT-PCR analysis of senescence-associated gene expression in vertebrae ($n = 4$ per group). (I) qRT-PCR analysis of TXN2 mRNA expression in vertebrae ($n = 4$ per group). (J) Immunoblot analysis of p21, p53, ARG2, TXN2, p62, and LC3 protein expression. Quantification of protein levels is shown in the accompanying bar graphs. (K) Representative images of calcein double labeling in trabecular bone and quantitative analysis of the mineral apposition rate (MAR). Scale bar = 50 μm. Statistical significance was assessed by one-way ANOVA. Data are presented as mean ± SEM. ns, not significant; $^{*}p < 0.05$, $^{**}p < 0.01$, $^{***}p < 0.001$ between the indicated groups.

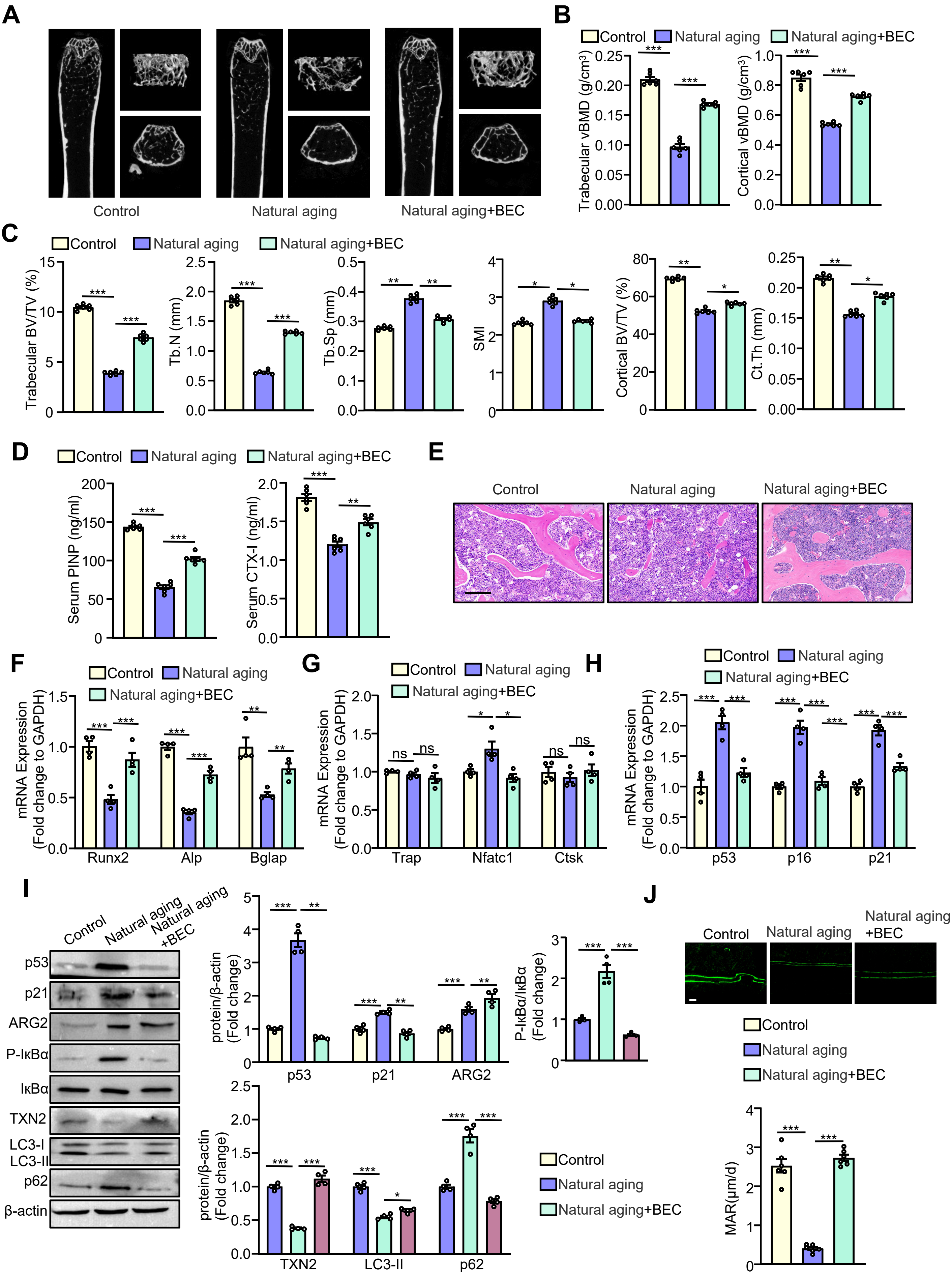

A
Control
Natural aging
Natural aging+BEC
B
Control
Natural aging
Natural aging+BEC
Trabecular vBMD (g/cm³)
Cortical vBMD (g/cm³)
C
Trabecular BV/TV (%)
Tb.N (mm)
Tb.Sp (mm)
SMI
Cortical BV/TV (%)
Ct.Th (mm)
D
Serum PINP (ng/ml)
Serum CTX-I (ng/ml)
E
F
mRNA Expression (Fold change to GAPDH)
Runx2
Alp
Bglap
G
Trap
Nfatc1
Ctsk
H
p53
p16
p21
I
p53
p21
ARG2
P-IκBα
IκBα
TXN2
LC3-I
LC3-II
p62
β-actin
protein/β-actin (Fold change)
P-IκBα/IκBα (Fold change)
LC3-II
J
Natural aging +BEC
MAR(μm/d)

Supplementary Figure S14. BEC ameliorates naturally aging-induced osteoporosis in mice. Naturally aged mice (20 months old) and age-matched young control mice (3 months old) were maintained for 18 weeks. Beginning at week 6, mice in the aging + BEC group received daily subcutaneous injections of BEC (2.3 mg/kg/day) for the remaining 12 weeks, whereas control mice received physiological saline for the same period. (A) Representative micro-computed tomography (micro-CT) images of vertebrae from young control mice, naturally aged mice, and naturally aged mice treated with BEC (n = 6 per group). (B) Quantitative analysis of trabecular and cortical volumetric bone mineral density (vBMD). (C) Quantitative micro-CT assessment of trabecular bone volume fraction (BV/TV), cortical BV/TV, structural model index (SMI), trabecular number (Tb.N), trabecular separation (Tb.Sp), and cortical thickness (Ct.Th) (n = 6 per group). (D) Serum levels of the bone turnover markers PINP and CTX-I measured by ELISA (n = 6 per group). (E) Representative H&E-stained images of decalcified vertebral sections from the indicated groups (n = 6 per group). Scale bar = 500 μm. (F) qRT-PCR analysis of osteoblast-related gene expression in vertebral tissues (n = 4 per group). (G) qRT-PCR analysis of osteoclast-related gene expression in vertebral tissues (n = 4 per group). (H) qRT-PCR analysis of senescence-associated gene expression in vertebral tissues (n = 4 per group). (I) Immunoblot analysis of p21, p53, ARG2, IκBα, phosphorylated IκBα (p-IκBα), TXN2, p62, and LC3 protein expression. Quantification of protein levels is shown in the accompanying bar graphs. (J) Representative images of calcein double labeling in trabecular bone. Scale bar = 50 μm. Quantification of the mineral apposition rate (MAR) is shown. Statistical significance was determined by one-way ANOVA. Data are presented as mean ± SEM. ns, not significant; *p < 0.05, **p < 0.01, ***p < 0.001 between the indicated groups.

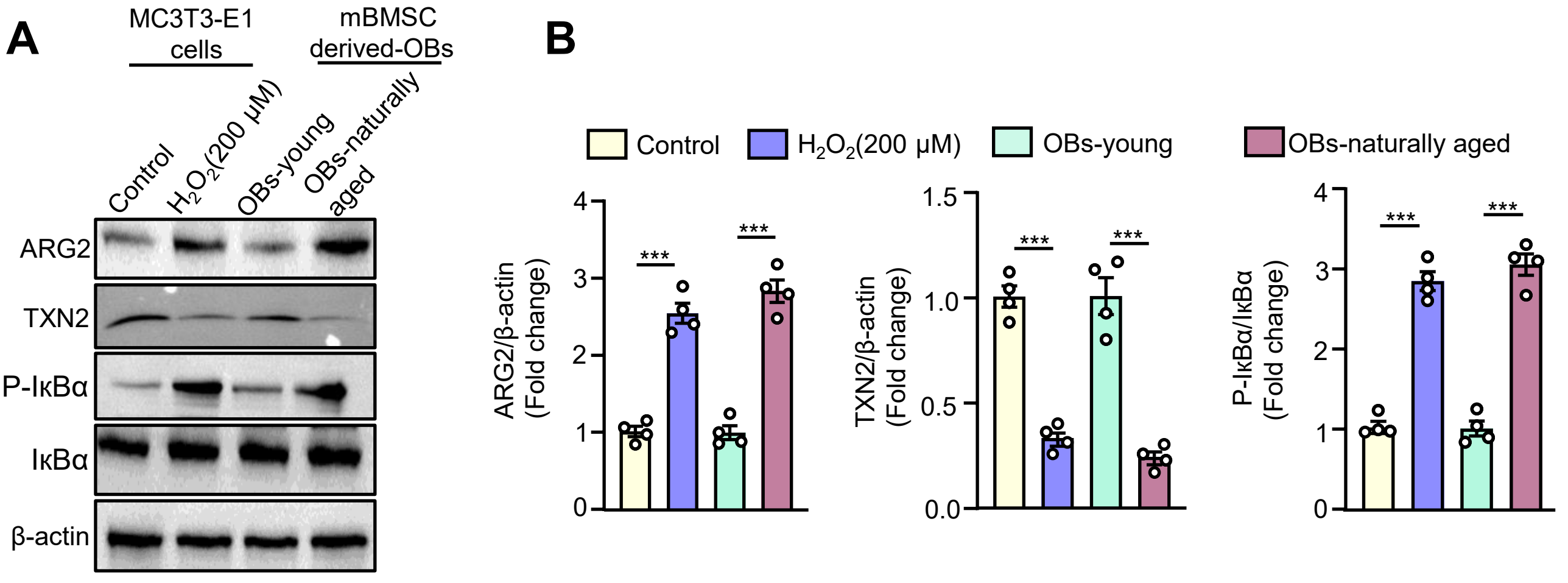

Supplementary Figure S15. Activation of the ARG2/IκBα–p65/TXN2 signaling axis in H_2O_2-induced senescent MC3T3-E1 cells and primary BMSC-derived osteoblasts from naturally aged mice. MC3T3-E1 cells were treated with H_2O_2 (200 μM) for 24 h to induce cellular senescence. Mouse bone marrow mesenchymal stem cells (mBMSCs) were isolated from the femurs of naturally aged mice (20 months old; OBs-naturally aged) and age-matched young mice (3 months old; OBs-young), followed by osteogenic differentiation. (A) Representative immunoblotting images showing the protein expression levels of ARG2, TXN2, phosphorylated IκBα (P-IκBα), and total IκBα. (B) Quantitative analysis of protein expression levels. Statistical significance was determined by one-way ANOVA. Data are presented as mean ± SEM (n = 4). ***$p < 0.001$ between the indicated groups.
